# Mixture-of-Experts Learning for Mixture-Response Interpretation and Screening of PE-ECC

**DOI:** 10.3390/ma19163363

**Published:** 2026-08-07

**Authors:** Yujie Wang, Lingzhi Li

**Affiliations:** College of Civil Engineering, Tongji University, Shanghai 200092, China; lilingzhi@tongji.edu.cn

**Keywords:** PE-ECC, mixture-of-experts learning, mechanical property prediction, mixture-response interpretation, support-filtered virtual screening

## Abstract

**Highlights:**

- A PE-ECC database comprises 383 material-level records from 90 literature sources.
- The MoE model predicts four PE-ECC properties with R^2^ values of 0.950–0.971.
- SHAP, ALE and response maps distinguish strength trends from tensile deformation.
- Binder, W/B, S/B and fiber variables show property-specific relationships.
- Support-filtered screening yields database-supported candidates for laboratory validation.

**Abstract:**

Featuring considerable tensile ductility and multiple cracking behavior, polyethylene fiber-reinforced engineered cementitious composites (PE-ECCs) are promising cement-based materials for engineering construction. However, establishing accurate design models for evaluating the mechanical properties of PE-ECC is a challenging task owing to the complex material components. This study presents an interpretable data-driven framework for predicting the mechanical properties of PE-ECC using mixture-of-experts (MoE) learning. A database comprising 383 deduplicated material-level records from 90 verified literature sources was compiled for modeling the compressive strength, ultimate tensile strain, ultimate tensile strength and first-cracking tensile strength of PE-ECC. An MoE prediction model was developed by integrating XGBoost, LightGBM, CatBoost, WDBPANN and TabPFN through out-of-fold stacking and learned gating. The model achieved coefficient of determination (*R*^2^) values of 0.971, 0.950, 0.970 and 0.954 for the four mechanical properties, respectively. Shapley additive explanations (SHAP), accumulated local effects (ALE) and response maps were used to examine the fitted nonlinear associations between the reported mixture variables and each target property. Based on these relationships, support-filtered virtual screening was conducted within the database-supported design space to identify candidate mixtures for subsequent experimental verification. The framework links target-specific prediction with mixture-response interpretation and confines screening to regions supported by reported PE-ECC mixtures.

## 1. Introduction

Engineered cementitious composites (ECCs) are strain-hardening cementitious materials designed to maintain tensile load after matrix cracking through fiber bridging and distributed multiple cracking. Compared with ordinary concrete, ECC shows higher tensile deformability, tighter crack-width control and better damage tolerance, which makes it attractive for repair and strengthening, link slabs, high-ductility structural components, 3D-printable cementitious materials and durability-oriented construction systems [1,2,3,4,5,6,7,8]. Polyethylene fiber-reinforced ECC (PE-ECC) has received particular attention because PE fibers provide high tensile strength, chemical stability and strong crack-bridging potential. For PE-ECC, however, increasing matrix strength alone does not guarantee stable tensile strain hardening. A denser matrix may increase compressive strength and first-cracking stress, but continued load transfer after cracking also depends on the fibers being well distributed and able to bridge successive cracks. The PE-ECC mixture design must therefore balance matrix strength, crack initiation, post-cracking resistance and tensile deformation.

These four properties cover complementary engineering requirements: compressive capacity, crack initiation, post-cracking tensile resistance and deformation through multiple cracking. Considering them together is useful when screening PE-ECC for repair, strengthening and other high-ductility applications, where strength and deformation compatibility must be balanced.

Reliable prediction of PE-ECC mechanical properties is important for reducing trial-and-error mixture design and for selecting promising mixtures before laboratory testing. This prediction task is difficult because the reported mechanical properties are governed by several coupled mixture variables. Binder composition and W/B affect hydration, pore structure, matrix strength and cracking resistance. S/B changes the matrix skeleton and the amount of paste available for fiber dispersion. Fiber volume, length, diameter, modulus and tensile strength describe the reported fiber parameters, but the resulting tensile behavior also depends on fiber orientation, dispersion and fiber–matrix interaction [9,10,11,12,13,14,15,16,17]. Empirical equations are usually too restrictive for such a heterogeneous mixture space. Micromechanical models provide the fundamental design criteria for ECC, including the stress and energy conditions for steady-state multiple cracking, but their direct use requires pullout laws, matrix fracture toughness, fiber orientation, crack spacing and crack-width information [1,2,3,13,14,15,16]. In the literature-derived PE-ECC datasets, these micromechanical quantities are usually not reported together with mixture proportions. Accordingly, the present analysis is built on the information that is consistently available across published PE-ECC studies: reported mixture proportions, fiber parameters and the main mechanical test results.

Data-driven modeling in civil engineering materials increasingly combines property prediction with interpretation. Shapley additive explanations (SHAP) and accumulated local effects (ALE) are used to identify influential variables and examine how fitted responses change across the observed range [18,19]. Recent ECC and PE-ECC studies also show that machine learning and deep learning models can represent coupled mixture–property relationships in heterogeneous experimental data [20,21,22]. White-box creator-variable methods offer another approach by constructing and selecting transformed predictors. HCVCM screens generated variables by target correlation and redundancy, whereas SVCM and its modified formulations use iterative generation and retention to strengthen the predictor set. Applications to concrete, artificial stone, hydrothermally solidified clay and UHPC have produced explicit predictive relations and improved accuracy [23,24,25,26]. The present study addresses a complementary problem by retaining the reported mixture variables so that binder closure and material meaning remain visible during interpretation. Tabular learning, stacking and adaptive expert combination are then used to handle the limited and heterogeneous PE-ECC data [27,28,29].

The materials-genome strategy for ECC has linked raw material attributes, mixture proportions, property prediction and inverse design [30]. SHCC multi-objective studies have further combined mechanical performance, environmental impact and material cost in mixture optimization [31]. Recent studies on 3D-printed ECC have combined material characterization with performance prediction using a back-propagation artificial neural network (BPANN) [32,33]. In the present ensemble, WDBPANN provides the neural-network learner alongside the tree-based and tabular models. Several gaps nevertheless remain for PE-ECC. Databases that mix different ECC or SHCC fiber systems may obscure trends specific to polyethylene fiber mixtures. First-cracking tensile strength is also reported and modeled less consistently than peak tensile strength or tensile strain, although it marks the transition from the uncracked matrix to post-cracking load transfer. In addition, feature-importance rankings alone do not show how fitted trends depend on binder closure, W/B and S/B, or how crack initiation differs from peak tensile resistance. Finally, prediction-based screening may enter poorly supported regions and is seldom checked against complete multi-response records before new candidates are proposed. The present study addresses these gaps using a PE-ECC database and separate MoE models for the four properties. It then examines their relationships with the reported mixture variables and restricts screening to the supported mixture space.

**Figure 1:**
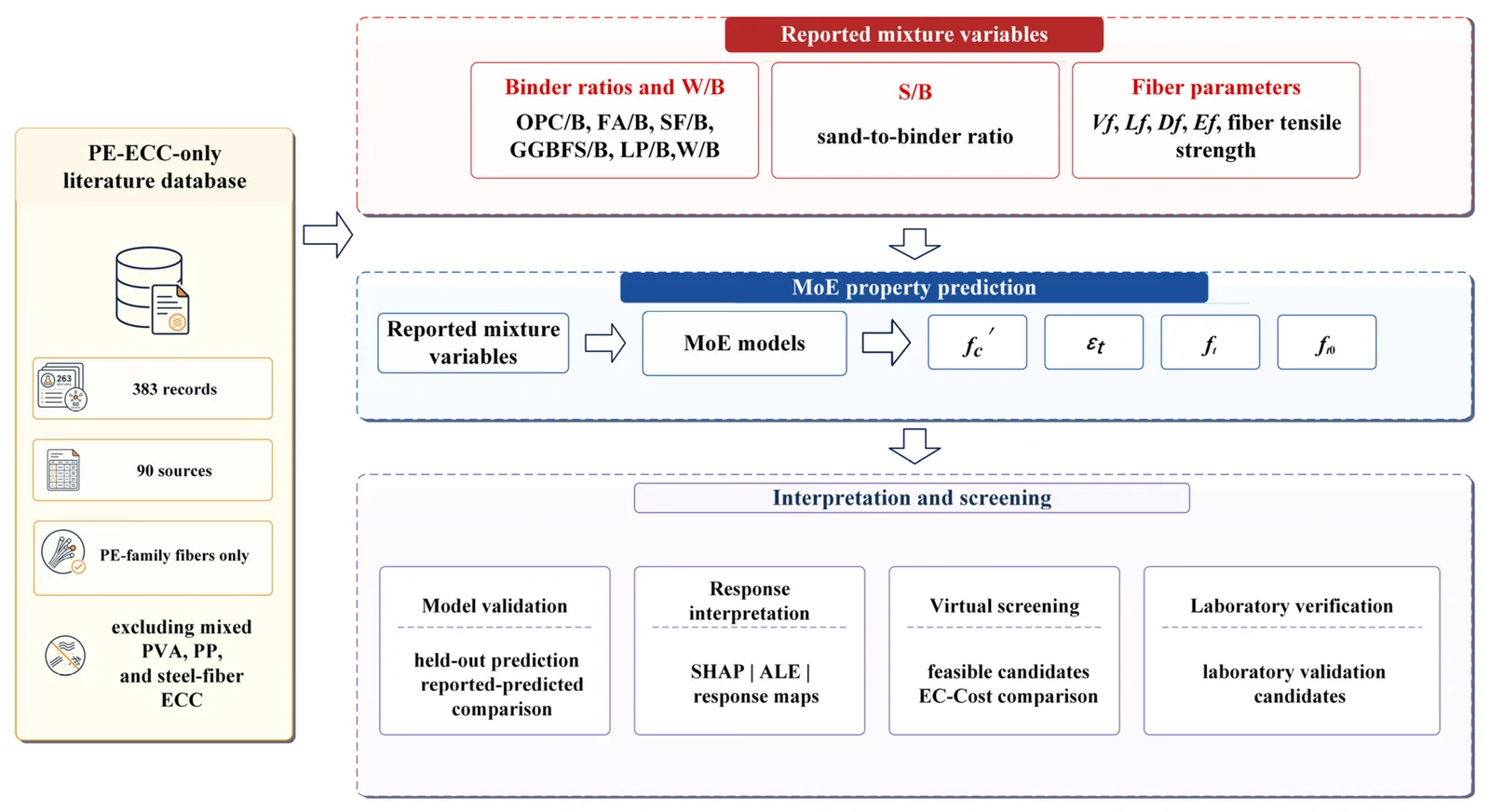
Workflow for PE-ECC property prediction, interpretation and mixture screening.

This study develops an interpretable MoE framework to predict PE-ECC mechanical properties, examine their relationships with the reported mixture variables and support mixture screening. A PE-ECC-only literature database was constructed from reported binder, sand and fiber variables. Target-specific MoE models were trained for compressive strength, ultimate tensile strain, ultimate tensile strength and first-cracking tensile strength by combining XGBoost, LightGBM, CatBoost, WDBPANN and TabPFN through stacking and learned gating. The fitted models were interpreted using SHAP, ALE, two-factor response maps and response surfaces to examine how binder composition, W/B, S/B and reported fiber parameters affect PE-ECC strength, tensile deformation and first cracking. The interpreted trends were then used for support-filtered virtual screening within the database-supported design space. The mechanically feasible candidates were compared using material-level embodied carbon (EC) and material cost (Cost) calculated from the literature factors. Figure 1 summarizes the workflow from PE-ECC database construction to MoE prediction, mechanism interpretation and mixture screening.

The specific contributions are:

- A PE-ECC-only database was assembled and four reported mechanical properties were modeled separately, so matrix strength, first cracking, peak tensile resistance and tensile deformation were examined as distinct material requirements.
- Five complementary learners were combined through out-of-fold stacking and learned gating. SHAP, ALE and Local Interpretable Model-agnostic Explanations (LIME) and response maps were then used to relate the fitted property trends to the reported binder, sand and fiber variables.
- Reported mixtures and virtual candidates were screened only within locally supported PE-ECC regions and compared using mechanical requirements, embodied carbon and material cost.

## 2. PE-ECC Database and Reported Mixture Variables

### 2.1. Database Scope and Target Responses

A PE-ECC-only database was assembled from published material-level records. The database was restricted to mixtures reinforced with polyethylene-family fibers, including PE and UHMWPE fibers, so that the learned trends were not mixed with those of PVA-, PP-, steel- or hybrid-fiber systems. Matrix-only mortars and component-level structural tests were also excluded. In this study, one material-level record refers to one PE-ECC mixture with reported mixture information and at least one target mechanical property.

The database contained 383 deduplicated material-level PE-ECC records from 90 verified literature sources [4,5,6,7,8,9,34,35,36,37,38,39,40,41,42,43,44,45,46,47,48,49,50,51,52,53,54,55,56,57,58,59,60,61,62,63,64,65,66,67,68,69,70,71,72,73,74,75,76,77,78,79,80,81,82,83,84,85,86,87,88,89,90,91,92,93,94,95,96,97,98,99,100,101,102,103,104,105,106,107,108,109,110,111,112,113,114,115,116,117]. Four target properties were considered: compressive strength ($f_{c}^{\prime }$), ultimate tensile strain (*ε_t_*), ultimate tensile strength (*f_t_*), and first-cracking tensile strength (*f_t_*_0_). The corresponding valid record numbers were 308, 368, 368 and 304, respectively. Missing target values were not filled by interpolation or by using other properties; each response model was trained only with records that reported the corresponding target. The complete PE-ECC dataset is provided in the Supplementary Materials (Data S1).

First-cracking tensile strength was extracted only from uniaxial tensile records. Reported first-cracking stress or strength values were used when they were tabulated directly. When the value was not tabulated, it was read from bilinear tensile models, reported average cracking stresses or clearly readable tensile stress–strain curves. Records that reported only peak tensile strength, calculated bridging stress, matrix cracking strength or flexural first-cracking strength were left without an *f_t_*_0_ value, rather than converted to uniaxial first-cracking tensile strength.

**Figure 2:**
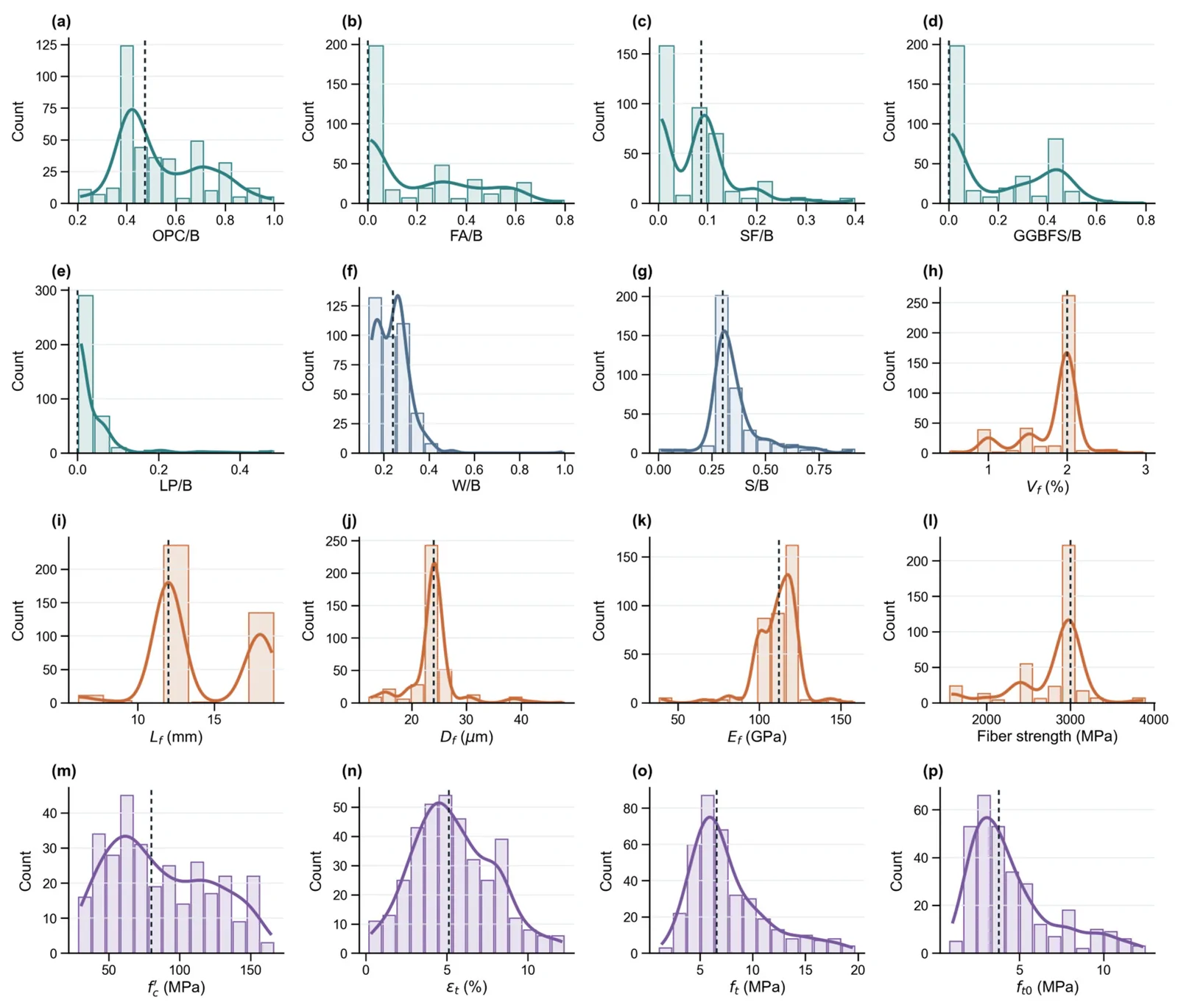
Distribution of reported mixture and fiber variables in the PE-ECC database: (**a**) OPC/B; (**b**) FA/B; (**c**) SF/B; (**d**) GGBFS/B; (**e**) LP/B; (**f**) W/B; (**g**) S/B; (**h**) *V_f_*; (**i**) *L_f_*; (**j**) *D_f_*; (**k**) *E_f_*; (**l**) fiber strength; (**m**) $f_{c}^{\prime }$; (**n**) *ε_t_*; (**o**) *f_t_*; (**p**) *f_t_*_0_. Bars and solid curves denote histogram counts and kernel density estimates, respectively; vertical dashed lines mark medians. Colors distinguish binder fractions, mixture ratios, fiber variables and target properties.

Figure 2 presents the statistical distribution of the input variables in the PE-ECC database. The collected mixtures do not uniformly cover the design space. Several SCM-related variables contain many zero values and are concentrated at several replacement levels, while W/B and S/B are distributed within relatively narrow practical ranges. The reported fiber parameters are also concentrated around commonly used PE-fiber configurations, indicating that non-standard fiber parameters are limited in both number and range. The later SHAP, ALE and two-factor plots therefore need to be read against this distribution. Trends in well-populated regions carry stronger evidence; sparse regions are treated as lower-support parts of the database.

### 2.2. Reported Mixture and Fiber Variables

The PE-ECC database provides information regarding the mixture proportions and fiber characteristics of each material-level record. Twelve reported variables were employed as model inputs, including OPC/B, FA/B, SF/B, GGBFS/B, LP/B, W/B, S/B, *V_f_*, *L_f_*, *D_f_*, *E_f_* and fiber tensile strength. These variables describe the relative proportions of cementitious components, water and sand contents relative to binder, and the reported dosage, dimensions and material properties of PE-family fibers.

According to the material-genome concept, the mechanical properties of ECC are closely related to both raw material attributes and mixture proportions. In the present literature-derived database, however, detailed raw material attributes were not consistently available. Therefore, the present study used the reported mixture and fiber variables as the material-level descriptors for model development and response interpretation.

For the normalized binder fractions used in the database, the cementitious components satisfy the following closure relation:

(1) OPC/B + FA/B + SF/B + GGBFS/B + LP/B = 1

Thus, OPC/B, FA/B, SF/B, GGBFS/B and LP/B were interpreted as a closed binder composition. A change in one binder fraction necessarily changes the balance of the remaining binder components. W/B and S/B were treated as ratio variables describing water availability and sand content relative to binder, respectively.

For the mechanism-oriented SHAP, ALE and two-factor analyses, the discussion mainly focused on ten mixture-design variables: OPC/B, FA/B, SF/B, GGBFS/B, LP/B, W/B, S/B, *V_f_*, *L_f_* and *D_f_*. *E_f_* and fiber tensile strength were retained as model inputs but were not emphasized in the SHAP discussion because their reported ranges were narrow and largely reflected differences among the PE-fiber products used in the source studies.

These reported variables were used in the target-specific MoE models described in Section 3.

## 3. MoE Learning Method and Model Validation

### 3.1. MoE Architecture and Learning Sequence

Four target-specific learning tasks were constructed for $f_{c}^{\prime }$, *ε_t_*, *f_t_* and *f_t_*_0_. All four models used the same 12 reported mixture and fiber variables. For each response, however, the training records were limited to mixtures that actually reported that response; a missing $f_{c}^{\prime }$, *ε_t_*, *f_t_* or *f_t_*_0_ value was not inferred from the other measured responses.

Missing target values were retained as missing. Missing input values were filled using the median from the same source where available and otherwise the database median. For each target, the available records were stratified by binned target values and divided into training and evaluation subsets at an approximately 8:2 ratio. The record-level split was repeated with different random seeds to examine sensitivity to record allocation.

Although the database contains 383 records, coverage is uneven across the 12 input variables because the records span different binder systems, sand contents, fiber configurations, curing conditions and test protocols. The base set comprised three gradient-boosting tree implementations, XGBoost, LightGBM and CatBoost [118,119,120,121], together with WDBPANN as the neural-network learner and TabPFN as the tabular foundation model [27].

The five base learners were selected before fitting to cover three model families: boosted trees, a neural network and a tabular foundation model. This compact set provides different regression structures without making the expert pool large relative to the PE-ECC database. The training-fraction analysis examines how the target-specific models respond to reductions in the available training records.

Each base learner generated out-of-fold predictions for the training records. These predictions were combined with base-learner disagreement and local-support descriptors as the inputs to the stacking layer. Four meta learners, Bayesian ridge, Huber regression, ElasticNet and a Huber-loss gradient-boosting regressor, were then combined by a learned softmax gate to obtain the final target-specific prediction. The Huber-based components were included to limit the influence of a small number of atypical observations on the stacking fit. The learned gate was used only for prediction. Each property model contained five base learners, four regularized meta learners and one gate; the meta learners acted only at the aggregation stage.

The target-specific MoE prediction is written as

(2) $$p_{i,k}^{\left(t\right)}={f_{k}}^{\left(t\right)}(x_{i}),\;k=1,\ldots ,K$$

(3) $$w_{i,r}^{\left(t\right)}=softmax\left[G^{\left(t\right)}\left(c_{i}\right)\right],\;{y_{i}}^{\left(t\right)}=\sum_{r=1}^{R}w_{i,r}^{\left(t\right)}m_{i,r}^{\left(t\right)},\;\sum_{r=1}^{R}w_{i,r}^{\left(t\right)}=1$$

In Equations (2) and (3), the base learners first provide target-specific predictions, and the meta-learner outputs are then combined by a softmax gate. All level-1 predictions used to train the meta learners and the gate were generated out-of-fold, so each training record was predicted by base learners that had not been fitted on that record. The same data-partition and evaluation procedure was applied to the reference models and the MoE model.

Local support was measured in the standardized feature space using k-nearest-neighbor distance, local density, Mahalanobis distance and the observed-envelope flag. These quantities entered the stacking layer and also identified predictions made in sparse parts of the database. Gate weights, disagreement and local-support descriptors were not used for material interpretation in Section 4.

Model complexity was constrained at both the expert and stacking levels. The tree learners used bounded depth, learning-rate control, row or feature sampling and regularization. WDBPANN used dropout, AdamW weight decay, gradient clipping and early stopping on an internal validation subset. Bayesian ridge and ElasticNet regularized the stacking coefficients, while the Huber-based meta learners reduced sensitivity to atypical residuals. Hyperparameters were selected separately for each response using Optuna (https://optuna.org/; accessed 6 May 2026) within the training protocol; the primary evaluation subset was not used to select a configuration. Together, these settings limit model variance and reduce sensitivity to individual records during repeated record-level evaluation.

Additional robustness calculations used three repeated five-fold outer splits generated with different random seeds. Records with identical reported 12-variable mixture signatures were kept in the same outer split. Within each training partition, the same out-of-fold base and meta-learning procedure was repeated using 40%, 60%, 80% and 100% of the initial training records; the outer held-out groups were not used for fitting or model selection. The same fixed model settings were retained at each training fraction, so these calculations served as robustness checks and did not replace the primary 8:2 evaluation in Table 1.

For model interpretation, Tree-SHAP was used to obtain a global importance pattern from the three tree-based base learners, whereas LIME was used to approximate complete MoE predictions within local mixture neighborhoods. For each target, 24 locally supported records spanning the reported response range were explained with 600 convex-interpolation samples drawn from their 40 nearest training mixtures. The interpolation retained the closed binder composition. Absolute LIME coefficients were averaged and normalized by target. Cosine similarity between the normalized LIME and Tree-SHAP importance vectors was used to assess agreement in the overall importance pattern, and the local surrogate *R*^2^ was retained as a fidelity measure. ALE profiles based on the final MoE predictions were then used to examine the direction and nonlinearity of the fitted relationships [18,19,122].

**Figure 3:**
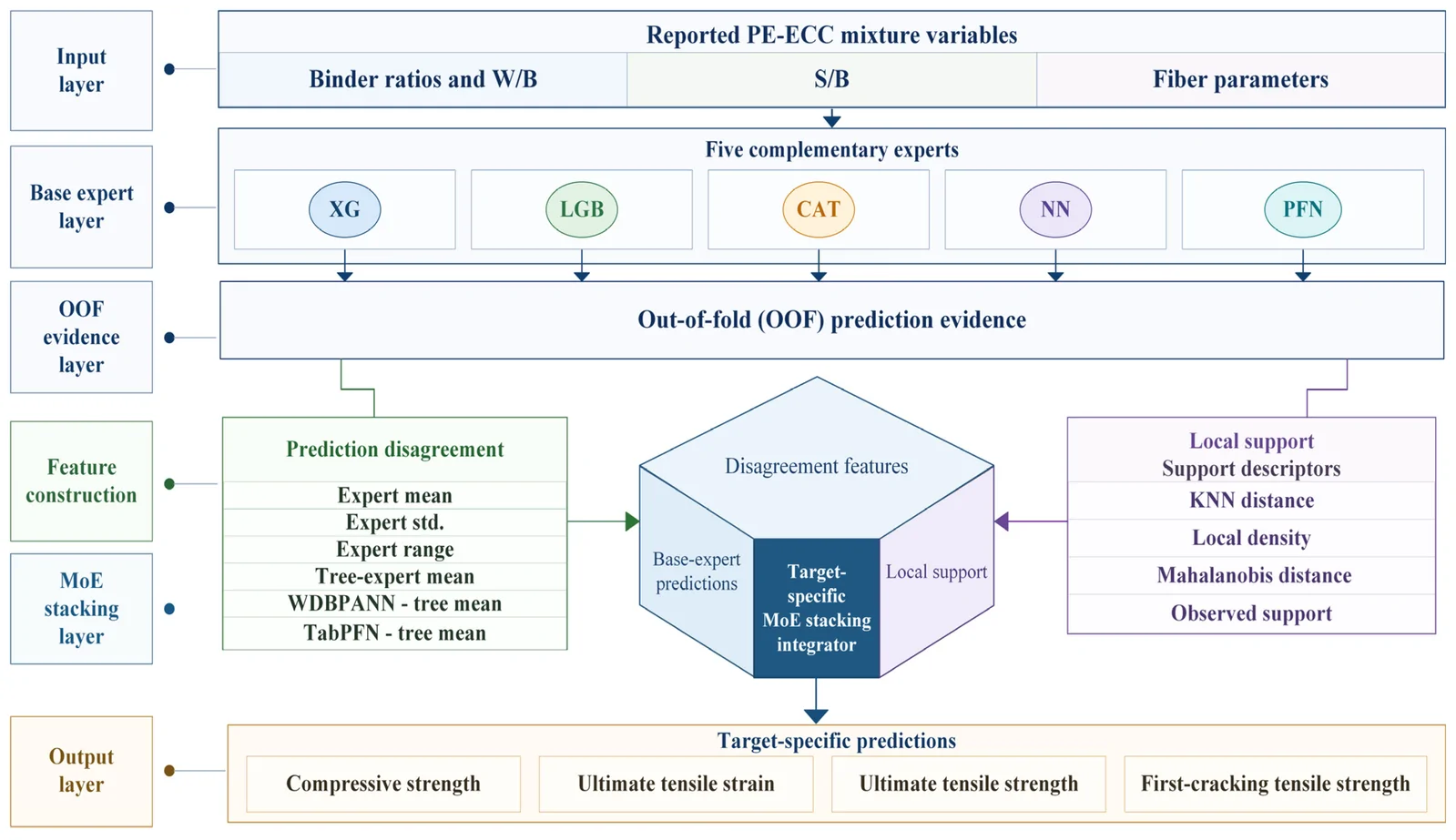
Architecture of the MoE stacking model for predicting the four PE-ECC properties.

Figure 3 summarizes the stacking sequence. Out-of-fold predictions from the five base learners were combined with disagreement and local-support descriptors, then passed to the target-specific meta-learning and gating layers for $f_{c}^{\prime }$, *ε_t_*, *f_t_* and *f_t_*_0_. The material interpretation in Section 4 uses the reported variables and fitted property trends rather than gate weights or support descriptors.

**Figure 4:**
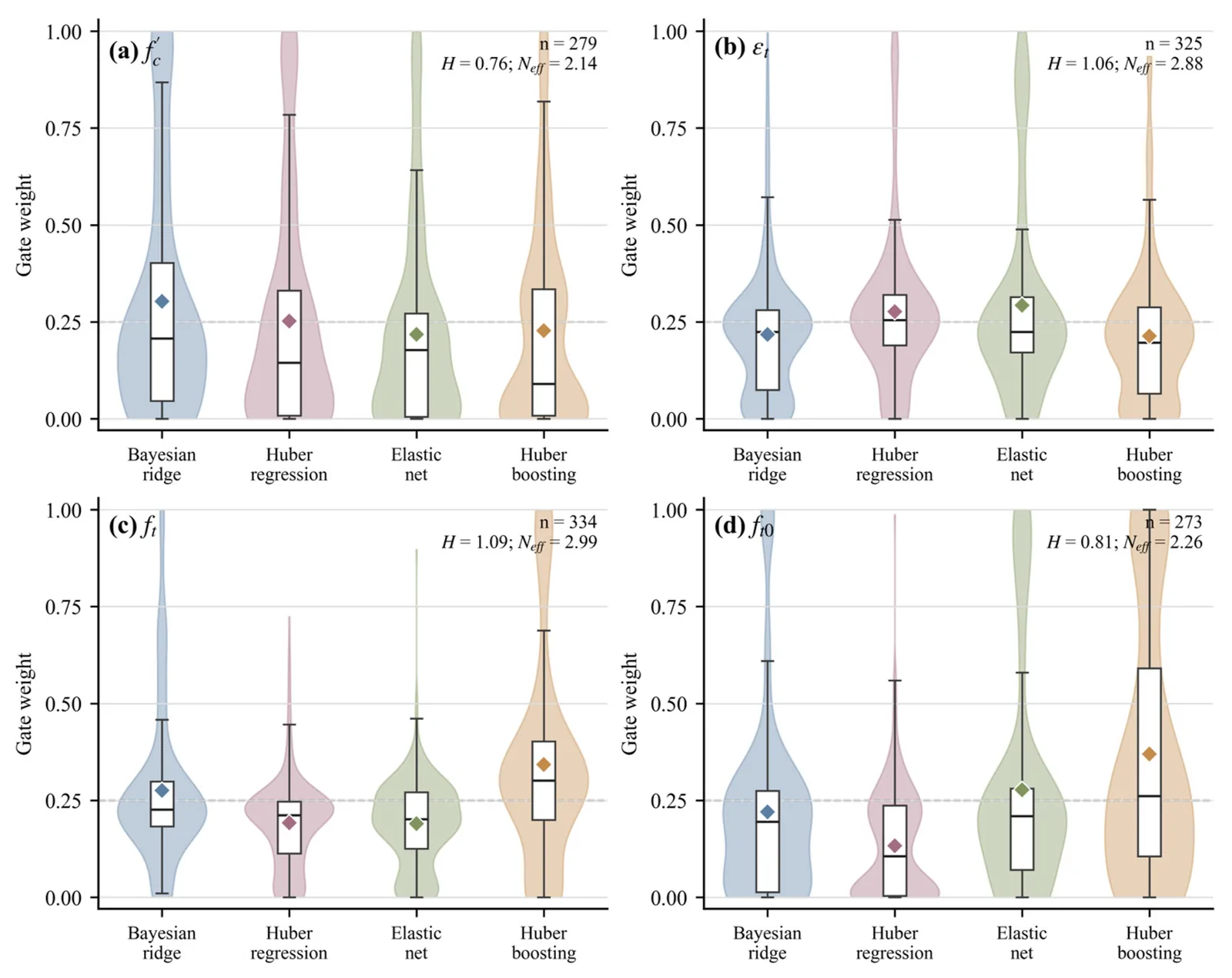
Distribution of learned gate weights across the four meta learners for the target-specific MoE models: (**a**) $f_{c}^{\prime }$; (**b**) *ε_t_*; (**c**) *f_t_*; (**d**) *f_t_*_0_.

Figure 4 shows the record-wise weights assigned by the learned gate. The mean gate entropy and effective number of contributing meta learners are defined as follows:

(4) $$H=-\frac{1}{N}\sum_{i=1}^{N}\sum_{j=1}^{4}w_{ij}ln(w_{ij}),\;N_{eff}=exp(H)$$

where N is the number of records and *w_ij_* is the weight assigned to meta learner j for record i. The weights vary across records, and the reported *N*_eff_ values indicate that each property model draws on more than one meta learner. The panels also show that the aggregation pattern differs among properties. Gate weights describe model combination, not material-variable importance.

### 3.2. Model Prediction Performance

The prediction performance of the target-specific MoE models was evaluated using out-of-fold validation during model development and the designated evaluation records for final reporting. The coefficient of determination (R^2^), root mean square error (RMSE), and mean absolute error (MAE) were adopted as statistical indicators. The values reported in Table 1 correspond to the evaluation records.

The performance indices of the four MoE models are listed in Table 1. The obtained R^2^ values for $f_{c}^{\prime }$, *ε_t_*, *f_t_*, and *f_t_*_0_ were 0.971, 0.950, 0.970, and 0.954, respectively. The corresponding RMSE/MAE values were 5.952/1.226 MPa, 0.562/0.305%, 0.585/0.307 MPa, and 0.449/0.239 MPa, respectively. Under the reported evaluation protocol, the MoE models reproduced the four reported PE-ECC properties with high accuracy.

To extend the comparison beyond the models used as MoE experts, LSBoost and GPR were included because both have been applied in recent performance-based ECC modeling, and TabNet was retained as a deep tabular reference [33,123]. All reference models were built using the same twelve input variables and followed the same train–test splitting strategy adopted for the full MoE model. Table 2 presents the coefficient of determination (R^2^) values obtained for each model and mechanical property.

Among the independent references, LSBoost was strongest for $f_{c}^{\prime }$ and *f_t_*, whereas GPR gave higher values for *ε_t_* and *f_t_*_0_. TabNet was less consistent across the four small-sample regression tasks. The MoE achieved the highest R^2^ for all four target properties, with the largest margin observed for *ε_t_*.

**Figure 5:**
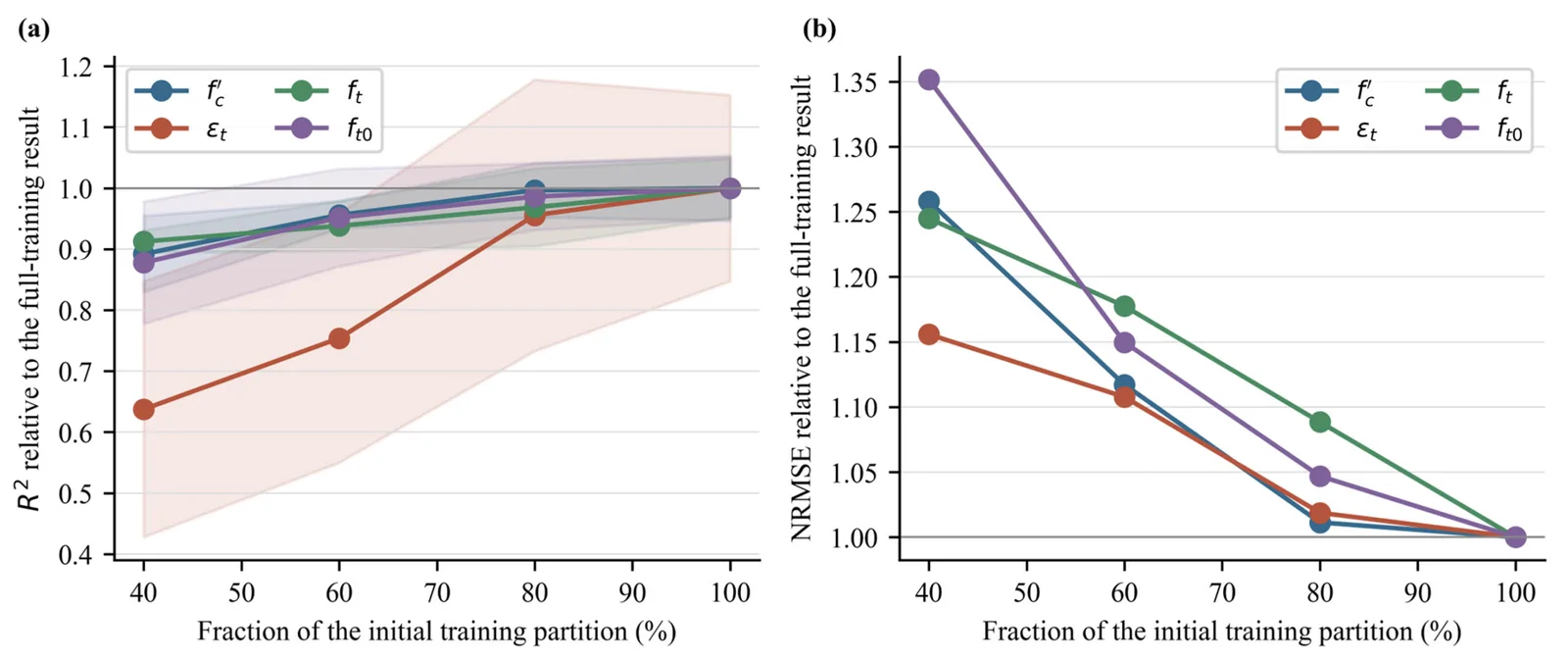
Training-fraction analysis for the target-specific MoE models: (**a**) *R*^2^ relative to the result obtained with the complete initial training partition; (**b**) NRMSE relative to the result obtained with the complete initial training partition. Lines show the mean across repeated mixture-disjoint diagnostic evaluations; shaded bands in (**a**) show one standard deviation.

Figure 5a,b show that the relative performance approaches the result obtained with all initial training records as the available training fraction increases. The tensile strain model has the widest variation at the smaller fractions, which is consistent with the more heterogeneous reported strain data.

**Figure 6:**
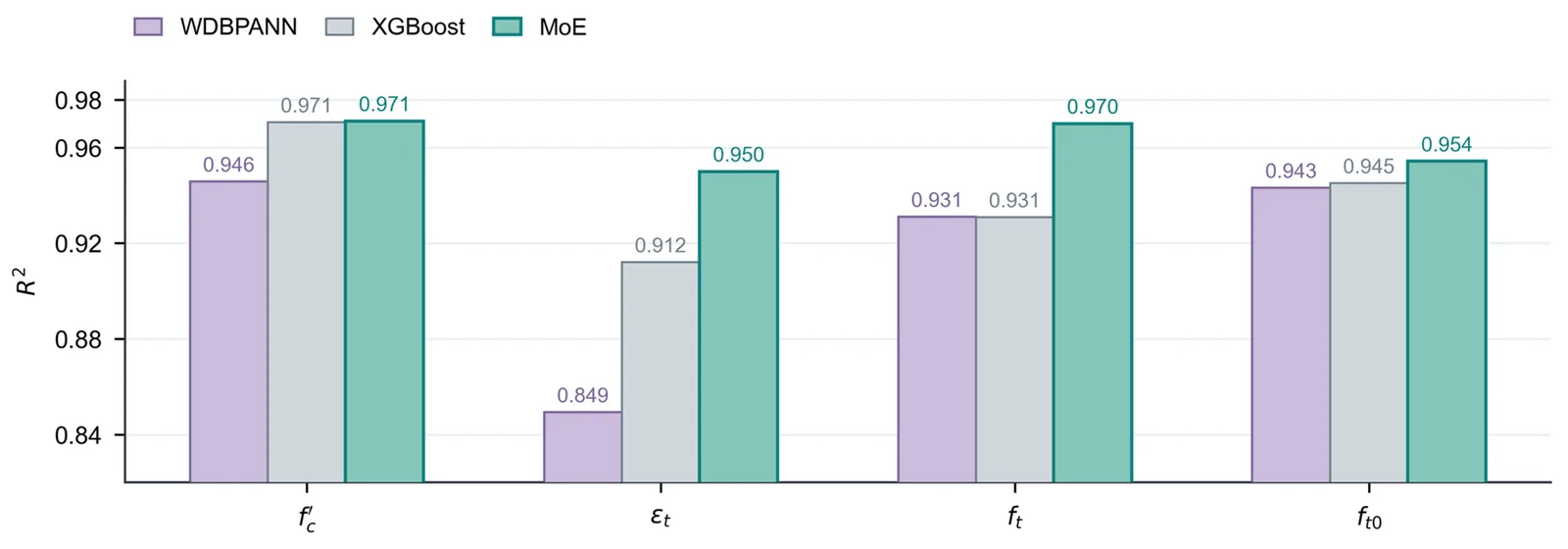
Prediction performance of the MoE model and the two reference models.

Figure 6 compares the MoE model with XGBoost and WDBPANN on the same evaluation records. They provide tree-based and neural-network reference results, respectively. The size of the difference between the MoE and the two reference models varies among the four targets.

The reference-model results are closer to the MoE result for $f_{c}^{\prime }$ and differ more for *ε_t_*, *f_t_* and *f_t_*_0_. Combining the five base learners reduces dependence on a single regression form and gives the larger tensile-target improvement shown in Figure 6. This comparison concerns predictive performance under the adopted evaluation protocol; the material trends are examined separately in Section 4.

**Figure 7:**
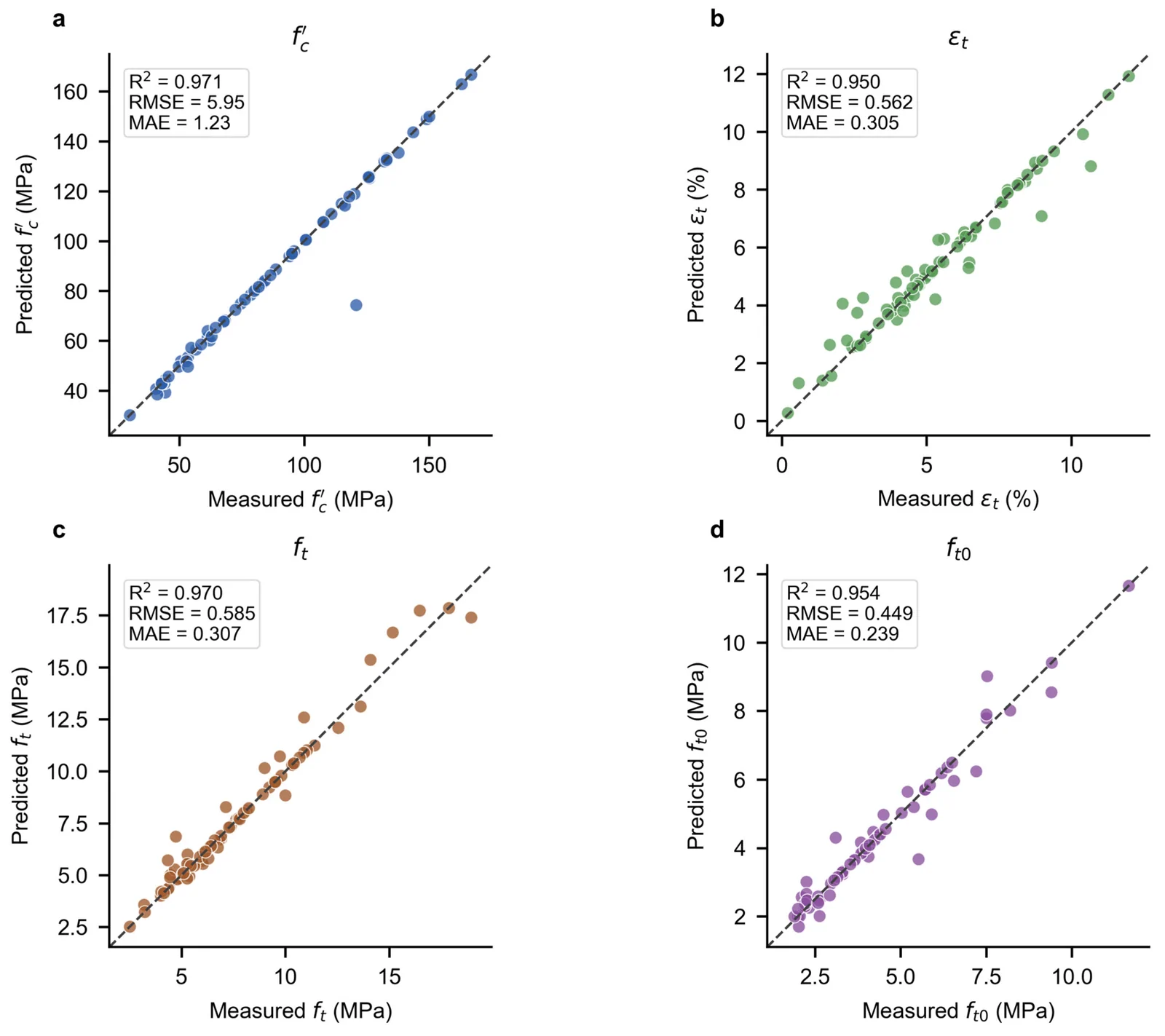
Reported and predicted values for the four properties: (**a**) $f_{c}^{\prime }$; (**b**) *ε_t_*; (**c**) *f_t_*; (**d**) *f_t_*_0_.

Figure 7 compares the reported and predicted values of the four PE-ECC properties. Most points lie close to the ideal prediction line, although the tensile-related targets show wider scatter than $f_{c}^{\prime }$. Together with the metrics in Table 1, these parity plots support analysis of fitted property trends within the reported PE-ECC range.

An independent case check used the recently reported M-C mixture with unmodified PE fiber [124]. Without refitting, the MoE predictions were close for *ε_t_* and *f_t_*, while $f_{c}^{\prime }$ was overestimated (Table 3). This single record is not treated as a statistical external-validation dataset.

## 4. Mechanism Analysis of PE-ECC Mixture-Response Relationships

### 4.1. Comparison of the Four PE-ECC Properties

A change in mixture composition does not affect the four properties in parallel. A direction that raises $f_{c}^{\prime }$ or *f_t_*_0_ may leave *f_t_* or *ε_t_* unchanged or reduce it. Section 4 therefore examines each fitted property trend before comparing their differences in Section 4.5.

ECC micromechanics provides the material basis for this interpretation. Stable multiple cracking requires sufficient fiber-bridging stress and complementary energy relative to the matrix cracking demand [1,2,3]. The present database was compiled from published PE-ECC mixture records, for which pullout curves, matrix fracture toughness, crack spacing and crack-width data are rarely available in a comparable form. The following analysis therefore remains at the mixture-response level and examines how reported binder, sand and fiber variables are associated with $f_{c}^{\prime }$, *ε_t_*, *f_t_* and *f_t_*_0_ within the collected PE-ECC range.

**Figure 8:**
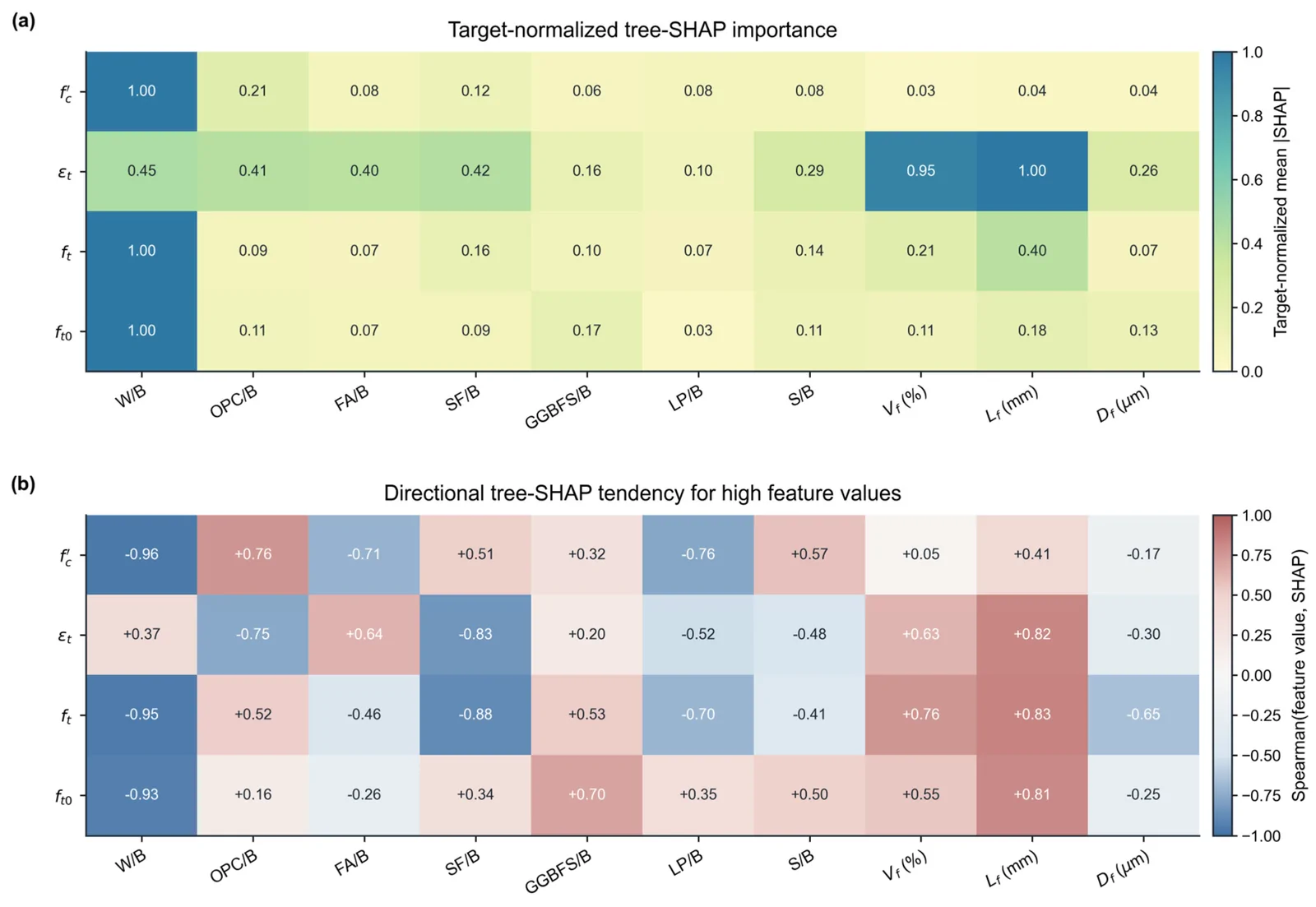
SHAP analysis of the reported mixture variables: (**a**) mean absolute SHAP values normalized within each property; (**b**) Spearman correlation between variable values and SHAP values.

Figure 8 summarizes the SHAP importance of the reported mixture variables for the four properties. The rankings differ among the targets. For $f_{c}^{\prime }$, W/B ranks first, in agreement with the established influence of water content on matrix compactness and compressive strength [10,11,12]. The binder-fraction rankings are examined as compositional changes in Section 4.2.

For *ε_t_*, the important variables are distributed among W/B, binder fractions, S/B and the nominal fiber variables. Tensile ductility is not governed by matrix densification alone. Distributed cracking requires the matrix cracking resistance to remain compatible with the bridging capacity developed by the fibers. W/B and binder composition influence matrix properties and rheology, while *V_f_*, *L_f_* and *D_f_* specify the reported fiber dosage and dimensions. At fixed *V_f_*, smaller *D_f_* increases the nominal number of fibers available to cross a crack plane, whereas longer *L_f_* increases the embedment length and aspect ratio. The realized contribution also depends on fiber dispersion and orientation [1,2,3,13,14,15,16].

W/B, S/B and the nominal fiber variables rank prominently in both the *f_t_*_0_ and *f_t_* models, but their ALE profiles and two-factor maps differ. The following analysis examines where these two tensile-strength measures differ within the reported PE-ECC range.

**Figure 9:**
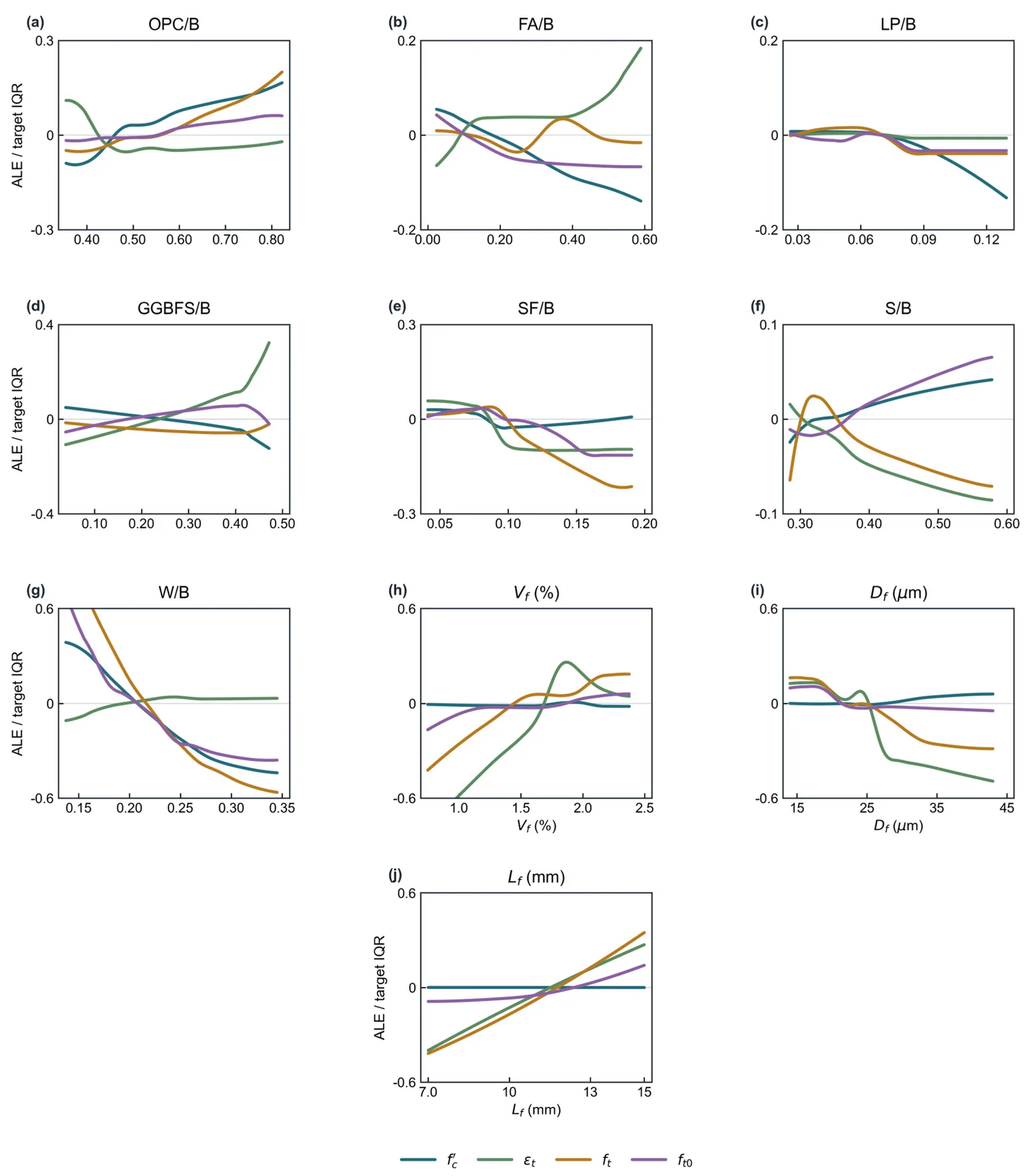
ALE profiles for the selected reported mixture variables: (**a**) OPC/B; (**b**) FA/B; (**c**) LP/B; (**d**) GGBFS/B; (**e**) SF/B; (**f**) S/B; (**g**) W/B; (**h**) *V_f_*; (**i**) *D_f_*; (**j**) *L_f_*.

Figure 9 shows the normalized ALE profiles of the ten mixture-design variables used in the mechanism-oriented analysis. The ALE values were normalized by the IQR of the corresponding target; therefore, the curves are used to compare the relative variation in the fitted property values within the database range rather than absolute increments in strength or strain. Together with the SHAP ranking in Figure 8, the ALE profiles provide information on the direction and nonlinearity of the mixture-response relationships.

Locally supported LIME explanations provided a local check on the global Tree-SHAP pattern. The cosine similarity between the normalized LIME and Tree-SHAP importance vectors was 0.965, 0.884, 0.938 and 0.916 for $f_{c}^{\prime }$, *ε_t_*, *f_t_* and *f_t_*_0_, respectively. The corresponding median local surrogate *R*^2^ values were 0.892, 0.784, 0.754 and 0.851. The agreement in the overall importance distributions supports the principal variable patterns identified by Tree-SHAP, while the lower local fidelity for *ε_t_* and *f_t_* reflects the greater difficulty of approximating the complete MoE response in those neighborhoods. Tree-SHAP summarizes the three tree learners over the database, whereas LIME approximates the complete MoE near individual records; the comparison is therefore used as supporting evidence rather than as a causal test.

The W/B profiles separate the strength-related properties from tensile ductility. The fitted values of $f_{c}^{\prime }$, *f_t_* and *f_t_*_0_ decrease as W/B increases, whereas *ε_t_* changes only slightly and non-monotonically. Lower W/B can produce a denser and stronger matrix, but distributed cracking also depends on the balance between matrix cracking demand and fiber-bridging capacity [1,2,3,10,11,12,13,16].

The binder-fraction profiles also differ among the four targets. Within the sampled range, the higher fitted values of $f_{c}^{\prime }$, *f_t_* and *f_t_*_0_ occur mainly at higher OPC/B, whereas the higher part of the *ε_t_* profile occurs at larger FA/B or GGBFS/B. SF/B and LP/B show no common monotonic trend across the four properties. Because the binder fractions are compositionally coupled, these profiles describe changes in the overall binder composition rather than isolated component effects.

Higher S/B is associated with larger fitted $f_{c}^{\prime }$ and *f_t_*_0_, while *ε_t_* and *f_t_* decline toward the upper end of the S/B range. Increasing S/B reduces the paste available around particles and fibers and can intensify particle–fiber interference, which may hinder uniform fiber distribution and distributed cracking [13,14,15,17].

The reported fiber parameters mainly influence the tensile properties. $f_{c}^{\prime }$ is nearly insensitive to *V_f_*, *L_f_* and *D_f_*, whereas *ε_t_*, *f_t_* and *f_t_*_0_ vary more clearly with these variables. Increasing *L_f_* is associated with higher fitted tensile values, and smaller *D_f_* is favorable for *ε_t_* and *f_t_*. At fixed *V_f_*, these changes increase the aspect ratio, embedment length and the nominal number of fibers intersecting a crack plane. The *V_f_* profile, especially for *ε_t_*, is not strictly monotonic; increasing fiber volume is beneficial only when matrix rheology permits adequate dispersion and the matrix cracking resistance remains compatible with fiber bridging [1,2,3,13,14,15,16].

Overall, Figure 9 indicates that the four PE-ECC properties show different mixture-response patterns. The strength-related properties are mainly associated with W/B, binder composition and matrix skeleton, whereas *ε_t_* is more sensitive to the combined effects of binder composition, S/B and nominal fiber dosage and geometry. Because these variables are coupled in an actual PE-ECC mixture design, the subsequent two-factor plots are used to examine whether the one-variable tendencies observed in Figure 9 remain valid under combined changes in mixture variables.

**Figure 10:**
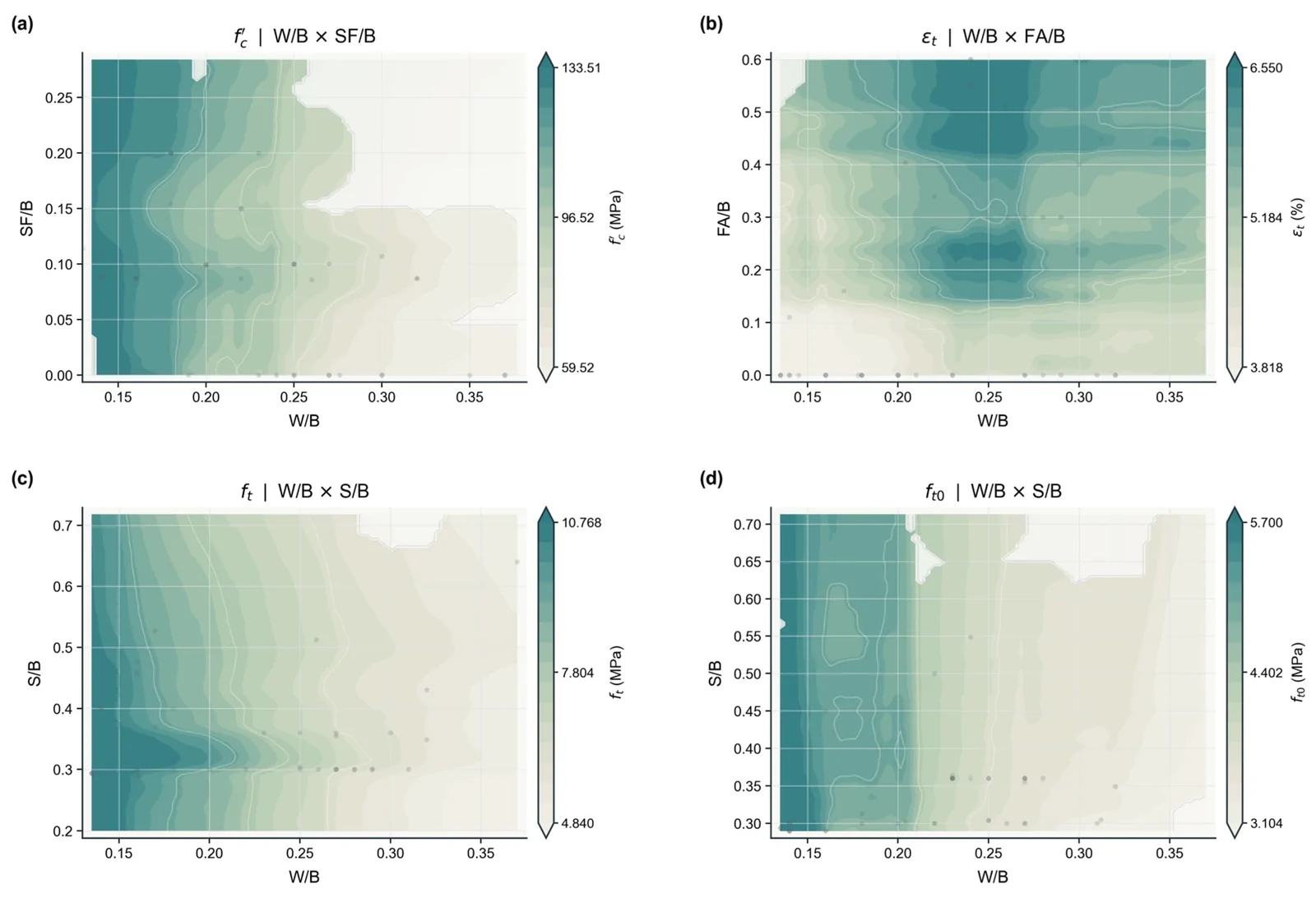
Response maps for selected pairs of reported mixture variables: (**a**) $f_{c}^{\prime }$ for W/B × SF/B; (**b**) *ε_t_* for W/B × FA/B; (**c**) *f_t_* for W/B × S/B; (**d**) *f_t_*_0_ for W/B × S/B.

Figure 10 shows four selected two-factor response maps based on the variables highlighted in Figure 9. The maps compare where higher predicted values of $f_{c}^{\prime }$, *ε_t_*, *f_t_* and *f_t_*_0_ appear within the collected PE-ECC mixture range.

For $f_{c}^{\prime }$, the W/B-SF/B map is mainly controlled by W/B. The higher-strength region remains at low W/B, while SF/B modifies the fitted strength level within this low-W/B range. This pattern is consistent with lower W/B increasing matrix compactness and silica fume contributing through particle packing and a pozzolanic reaction [10,11,12].

For *ε_t_*, the higher-strain region lies at moderate W/B and in FA-containing compositions rather than along the low-W/B band that gives the highest $f_{c}^{\prime }$. Distributed cracking requires the matrix cracking demand to remain below the available fiber-bridging capacity. A very dense matrix can raise that demand, whereas an excessively weak matrix may provide insufficient stress transfer; neither condition necessarily maximizes *ε_t_*. High-volume-FA ECC studies likewise associate FA-rich matrices with lower matrix cracking resistance and greater tensile ductility [1,2,3,11,13,16].

Both *f_t_* and *f_t_*_0_ remain higher toward low W/B, but their changes along S/B are not parallel. The S/B difference is examined in Section 4.3.

Each target in Figure 10 has its own color scale, so colors show variation within each property and are not directly comparable among properties. The following subsections examine the binder, sand and fiber variables in turn.

### 4.2. Effects of Binder Composition and W/B on Strength and Tensile Behavior

Lower W/B generally increases matrix compactness and reduces capillary porosity, which accounts for the strong W/B gradient in the strength maps [10,11,12]. Because the binder fractions in Equation (1) sum to unity, they are interpreted as a composition rather than as independent dosages. A greater GGBFS/B increases the slag proportion. Its latent hydraulic reaction can contribute to later-age matrix strength, while changes in fresh-state rheology may also affect fiber dispersion [10,124,125]. LP/B introduces a different balance: cement dilution can reduce matrix strength and cracking resistance, whereas particle packing and nucleation can improve compactness and early hydration [12,95,125].

**Figure 11:**
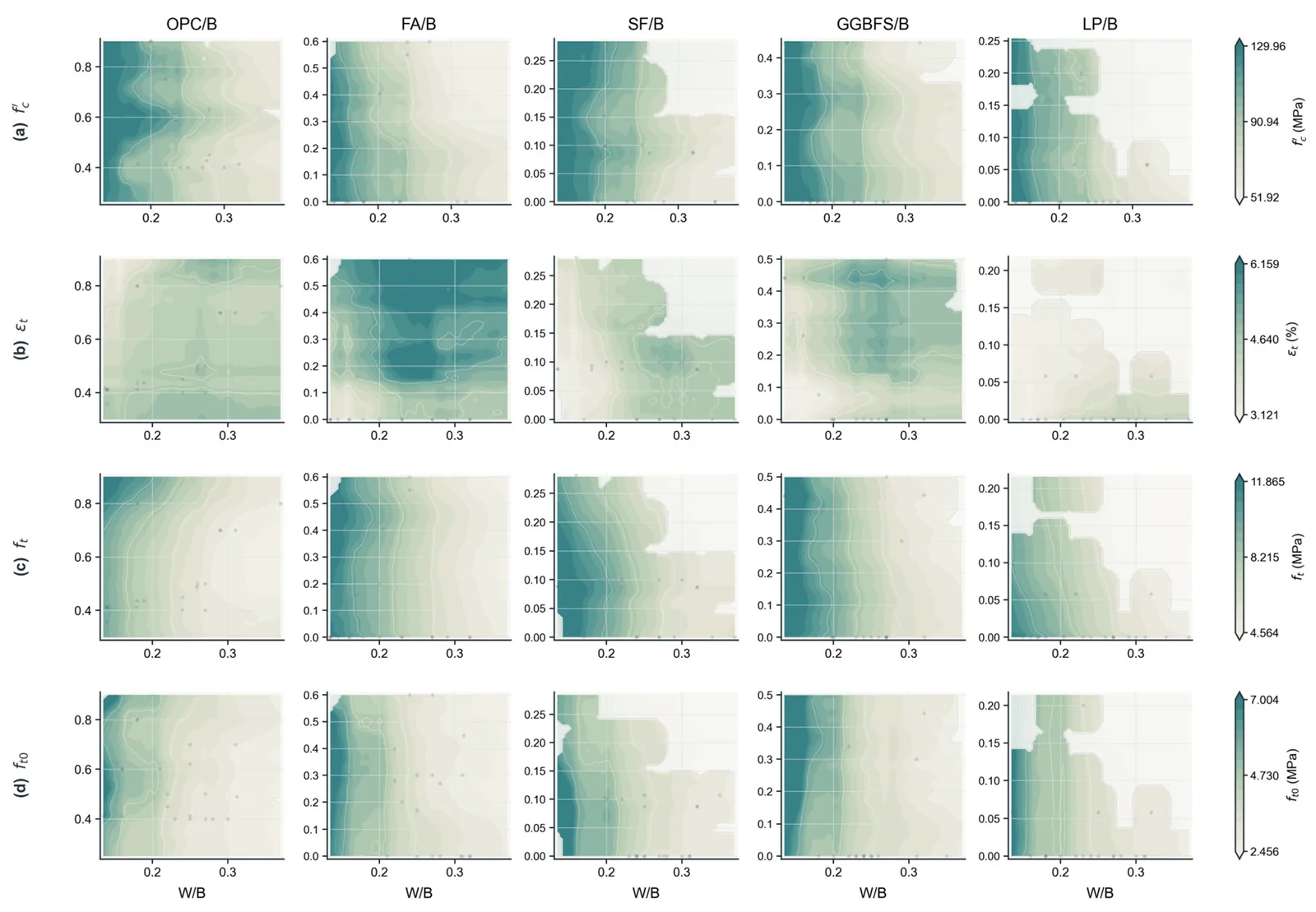
Response maps of W/B and binder composition. Rows show (**a**) $f_{c}^{\prime }$, (**b**) *ε_t_*, (**c**) *f_t_* and (**d**) *f_t_*_0_; columns show OPC/B, FA/B, SF/B, GGBFS/B and LP/B.

Figure 11 shows a low-W/B high-value region for $f_{c}^{\prime }$ across the selected binder planes. The higher *ε_t_* values occur at moderate W/B and in FA- or GGBFS-containing compositions, while *f_t_* and *f_t_*_0_ remain closer to the low-W/B region. The tensile strain region is displaced from the part of the mixture space associated with the highest matrix strength.

**Figure 12:**
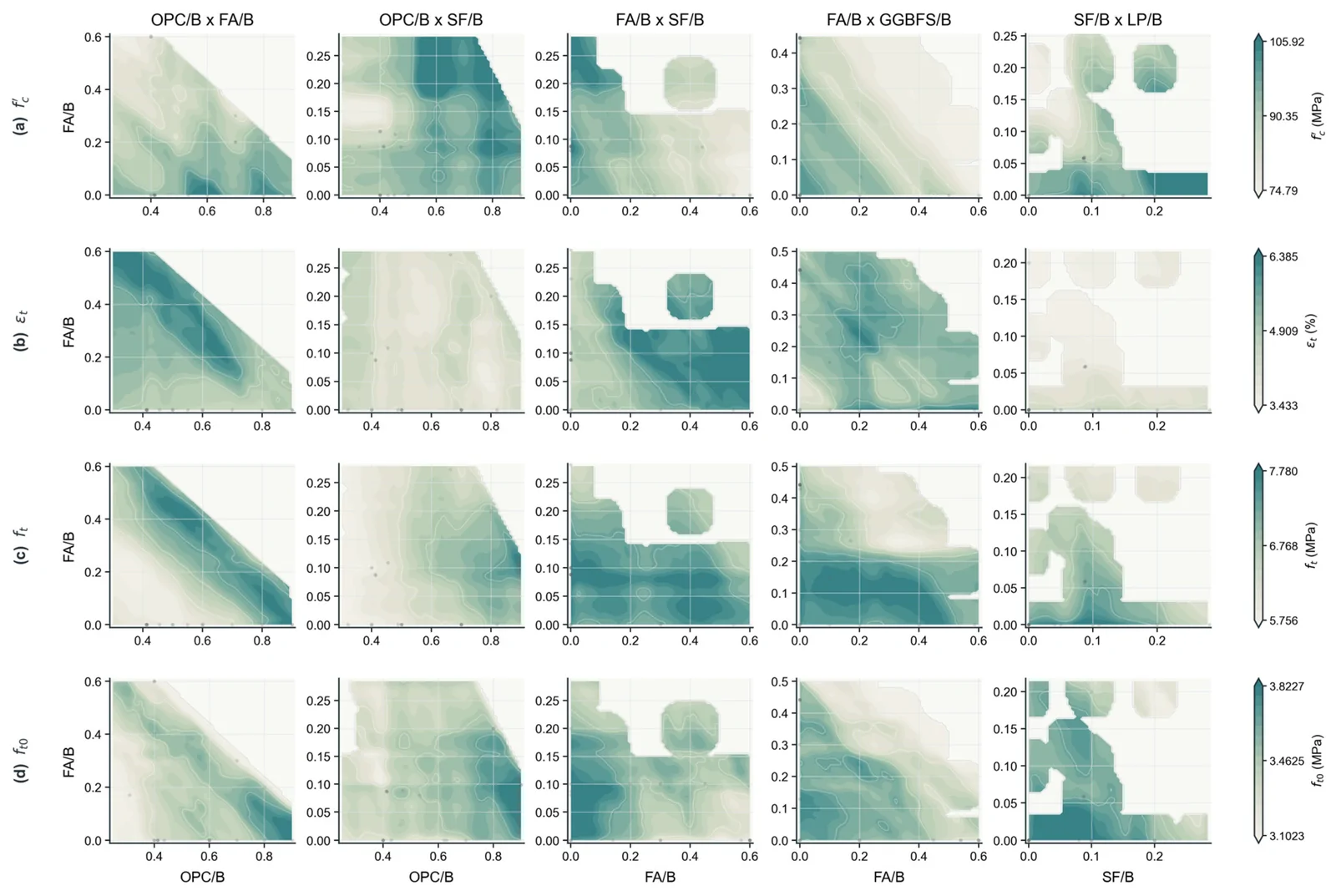
Response maps for pairs of binder fractions. Rows show (**a**) $f_{c}^{\prime }$, (**b**) *ε_t_*, (**c**) *f_t_* and (**d**) *f_t_*_0_; columns show OPC/B × FA/B, OPC/B × SF/B, FA/B × SF/B, FA/B × GGBFS/B and SF/B × LP/B.

Figure 12 shows pronounced composition gradients in the OPC/B-FA/B and FA/B-SF/B maps. Higher $f_{c}^{\prime }$, *f_t_* and *f_t_*_0_ occur more often in OPC-rich or SF-containing regions, whereas higher *ε_t_* appears more often in FA- or FA-GGBFS-containing regions. Because the binder fractions vary together, these trends refer to joint binder states rather than isolated component effects. The FA-GGBFS regions with higher *ε_t_* may reflect a balance among matrix cracking resistance, slag reaction and the fresh-state conditions needed for fiber dispersion [10,124,125]. The LP/B panels show localized high-value areas rather than a monotonic band, consistent with the competing effects of cement dilution, particle packing and nucleation [12,95,125,126].

**Figure 13:**
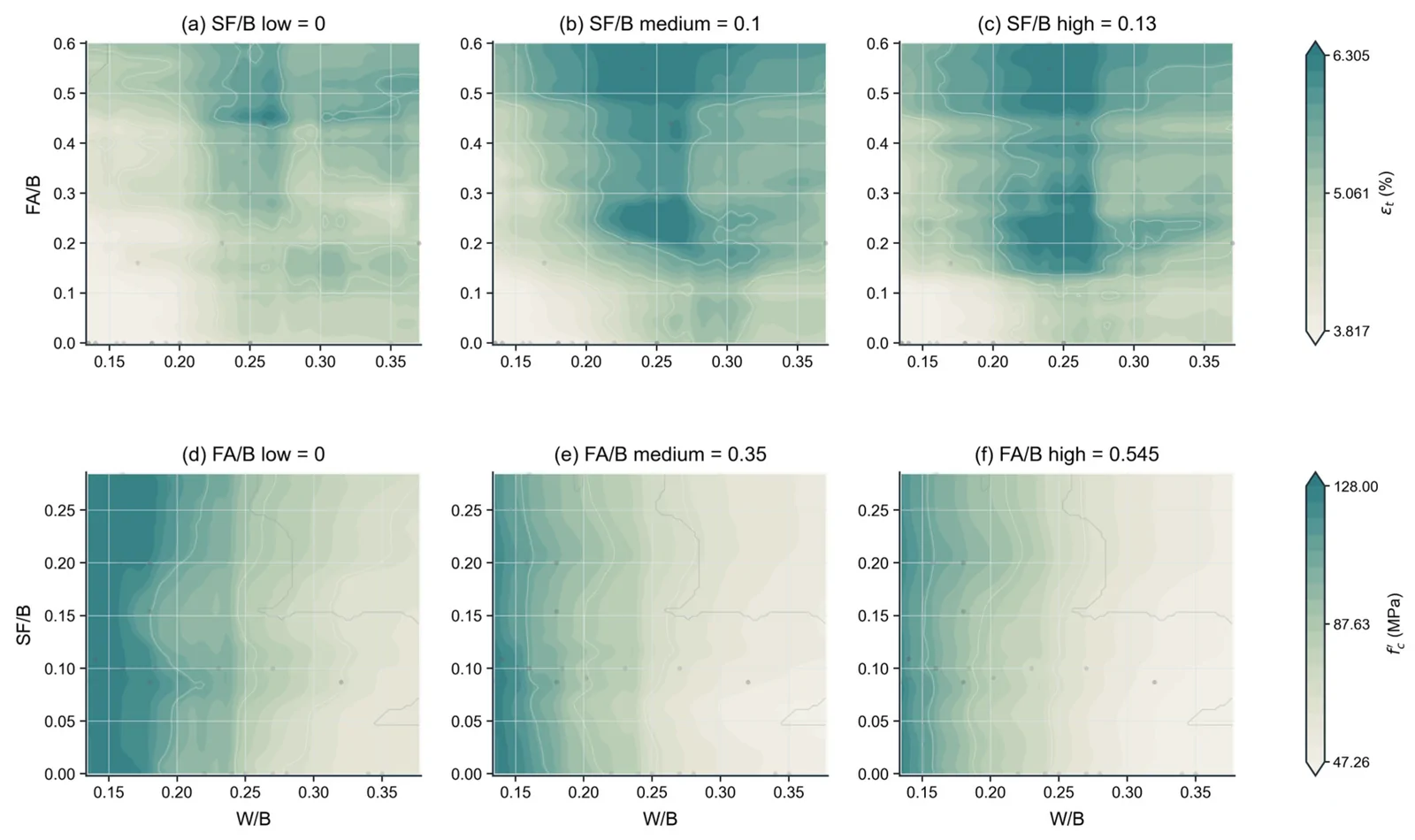
Predicted tensile strain and compressive strength at selected binder fractions: (**a**) *ε_t_* at SF/B = 0; (**b**) *ε_t_* at SF/B = 0.10; (**c**) *ε_t_* at SF/B = 0.13; (**d**) $f_{c}^{\prime }$ at FA/B = 0; (**e**) $f_{c}^{\prime }$ at FA/B = 0.35; (**f**) $f_{c}^{\prime }$ at FA/B = 0.545.

Figure 13 tests whether the W/B-FA/B and W/B-SF/B patterns persist as a third binder fraction changes. Across the *ε_t_* panels, higher values remain concentrated near moderate W/B and FA-containing compositions at the represented SF/B levels. Across the $f_{c}^{\prime }$ panels, varying FA/B changes the fitted magnitude without displacing the high-value region from low W/B. Taken with Figure 11 and Figure 12, the strength-related properties are concentrated mainly in low-W/B, OPC- or SF-rich regions, whereas higher *ε_t_* occurs more often in FA- or GGBFS-containing regions. The LP/B panels show no stable monotonic gradient over the sampled range. Section 4.3 next examines S/B and the reported fiber variables.

### 4.3. Effects of S/B and Fiber Parameters on Tensile Behavior

The following maps examine S/B together with the reported fiber dosage and dimensions, building on the binder-composition and W/B results above.

**Figure 14:**
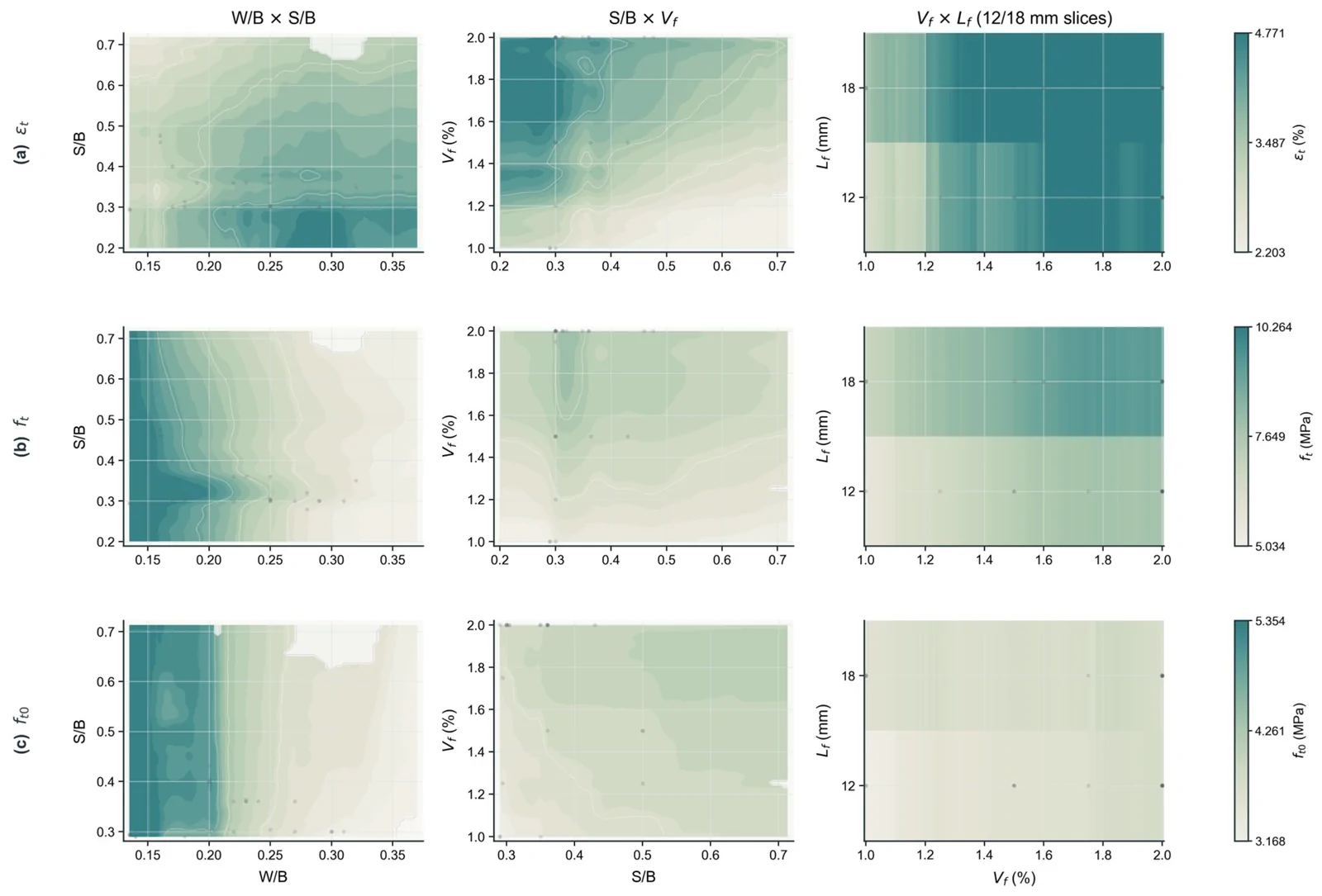
Response maps for S/B and reported fiber parameters. Rows show (**a**) *ε_t_*, (**b**) *f_t_* and (**c**) *f_t_*_0_; columns show W/B × S/B, S/B × *V_f_* and *V_f_* × *L_f_* using the reported 12 and 18 mm fiber lengths.

The W/B-S/B maps in Figure 14 place high *f_t_* mainly at low W/B and low-to-moderate S/B, whereas *f_t_*_0_ remains high across a broader low-W/B region. This difference is compatible with ECC micromechanics: first cracking depends primarily on the matrix cracking threshold, while peak tensile stress also depends on the bridging law after cracking [1,2,3,16].

For *ε_t_*, the higher values lie at moderate W/B and lower S/B, outside the region of the highest matrix strength. At lower S/B, the larger paste fraction and reduced particle–fiber interference may favor a more uniform fiber distribution [13,14,15,17]. In the S/B-*V_f_* map, the same region extends toward the higher-*V_f_* part of the supported range; *f_t_* and *f_t_*_0_ vary less sharply along S/B alone.

**Figure 15:**
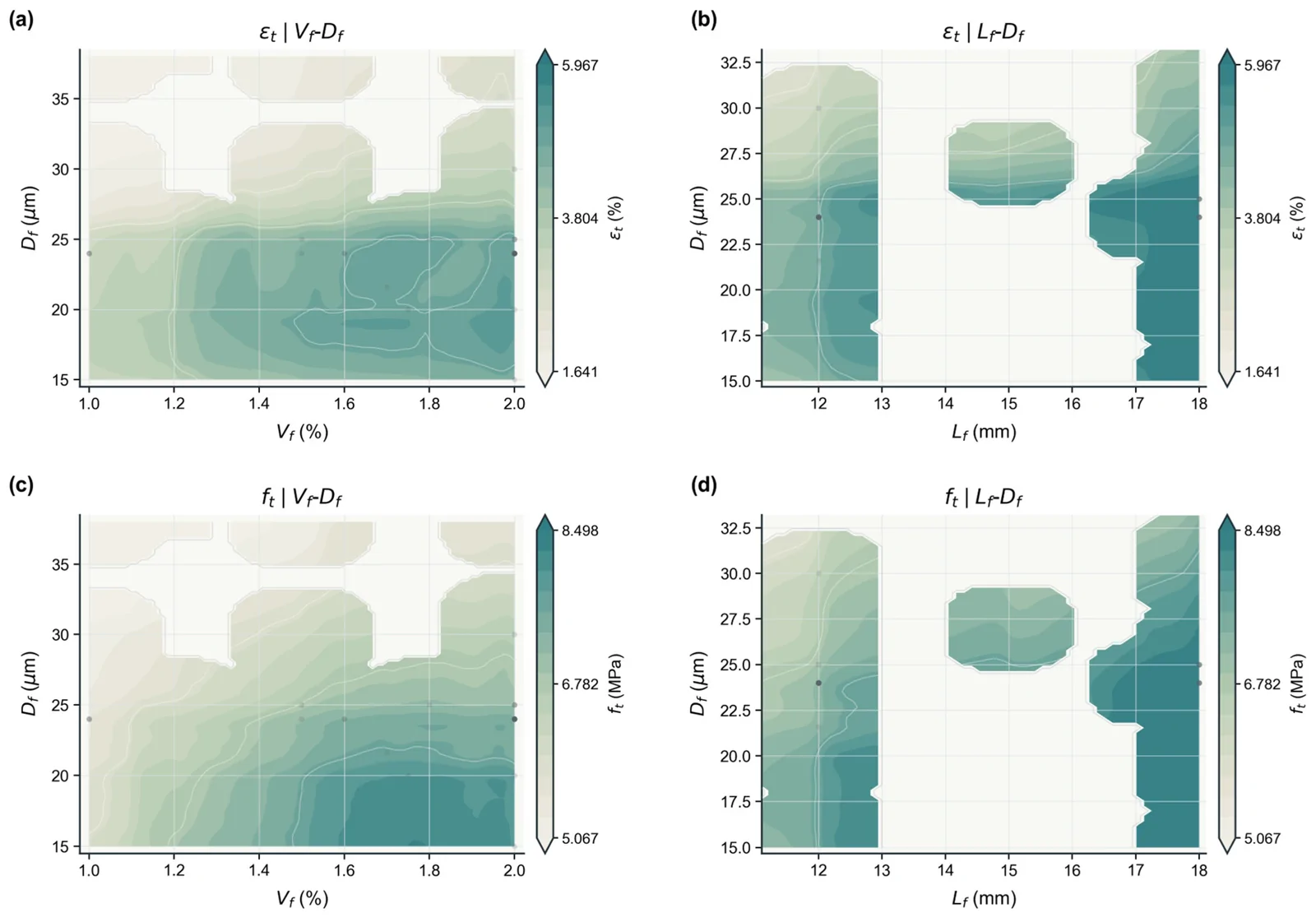
Response maps of tensile strain and peak tensile strength for the reported fiber dosage and dimensions: (**a**) *ε_t_* for *V_f_* × *D_f_*; (**b**) *ε_t_* for *L_f_* × *D_f_*; (**c**) *f_t_* for *V_f_* × *D_f_*; (**d**) *f_t_* for *L_f_* × *D_f_*.

Figure 15 focuses on *V_f_*, *L_f_* and *D_f_*. Higher fitted tensile values occur mainly at higher *V_f_*, longer *L_f_* and smaller *D_f_*. At fixed *V_f_*, smaller *D_f_* increases the nominal number of fibers that can intersect a crack plane, while longer *L_f_* increases the aspect ratio and embedment length; the actual bridging behavior remains dependent on orientation, dispersion and fiber–matrix interaction [3,13,14,15,16]. Taken with Figure 14, these maps show that the effects of fiber dosage and dimensions vary with S/B. This interaction motivates the *ε_t_*/FI analysis in Section 4.4.

### 4.4. Tensile Strain per Nominal Fiber Index and Post-Cracking Stress Increment

**Figure 16:**
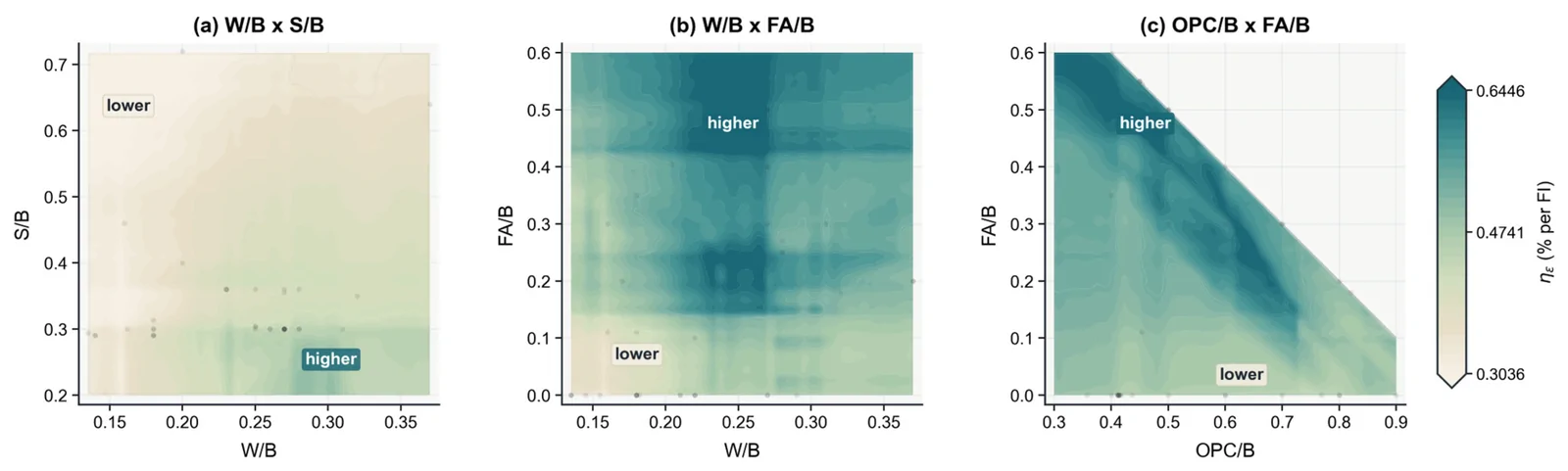
Tensile strain normalized by the nominal fiber index: (**a**) W/B × S/B; (**b**) W/B × FA/B; (**c**) OPC/B × FA/B.

Section 4.3 showed that *V_f_*, *L_f_* and *D_f_* are associated with the tensile properties. To compare tensile strain against their combined dosage and dimensions, Figure 16 normalizes *ε_t_* by a simple nominal fiber index:

(5) $$FI=\frac{V_{f}}{100}\frac{L_{f}}{D_{f}},$$

where *V_f_* is expressed in percent and *L_f_*/*D_f_* is the fiber aspect ratio. FI combines the reported fiber dosage and aspect ratio. The ratio *ε_t_*/FI is used to compare the tensile strain developed at a similar nominal fiber index; it is not a direct measure of fiber-bridging efficiency.

Figure 16 maps *ε_t_*/FI across the reported PE-ECC mixture space. Higher values occur mainly at lower S/B, moderate-to-high W/B and FA-containing binder compositions. This distribution shows that tensile strain relative to FI varies with matrix and sand proportions. FI represents the fiber dosage and aspect ratio, whereas matrix cracking resistance and the conditions governing fiber distribution also affect the strain developed at a given FI [13,14,15,16,17].

Two derived indicators were used to compare first cracking with peak tensile stress:

(6) $$\Delta f_{t}=f_{t}-f_{t0},\;\rho_{t}=\frac{f_{t}}{f_{t0}}$$

where Δ*f_t_* denotes the stress increase from first cracking to peak tensile strength, and *ρ_t_* is the corresponding strength ratio. These two indicators describe the reported tensile stress gain after the first crack and are used to compare *f_t_*_0_ and *f_t_* in the database.

**Figure 17:**
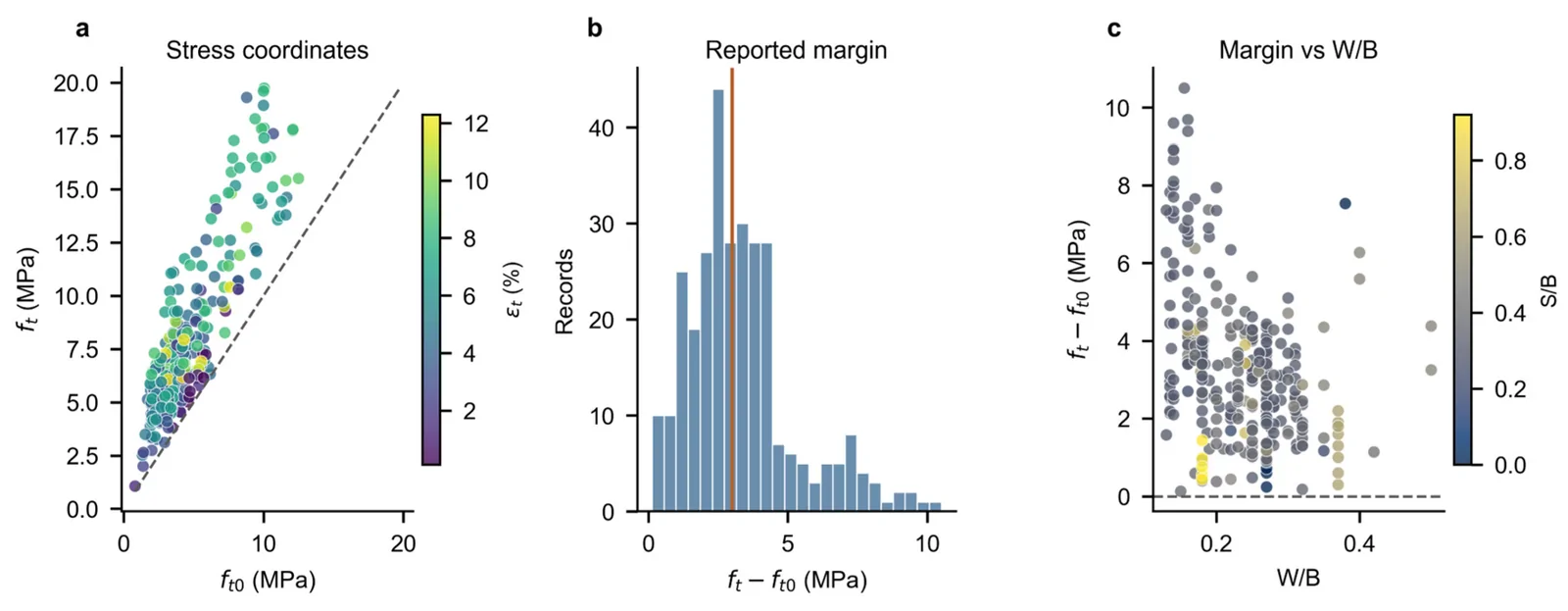
First-cracking and peak tensile stresses: (**a**) *f_t_* versus *f_t_*_0_, colored by *ε_t_*; (**b**) distribution of Δ*f_t_*; (**c**) Δ*f_t_* versus W/B, colored by S/B.

Figure 17 shows considerable variation in Δ*f_t_* among records with comparable *f_t_*_0_. The W/B coloring likewise shows no monotonic increase in Δ*f_t_* with *f_t_*_0_. This scatter is compatible with the two micromechanical conditions for strain hardening: crack initiation depends on the matrix cracking threshold, whereas stress development after cracking depends on the available bridging law and complementary energy [1,2,3].

Figure 16 and Figure 17 show that nominal fiber geometry and first-cracking stress alone do not determine the subsequent tensile behavior. Matrix and sand conditions affect the strain developed for a given FI, and mixtures with similar *f_t_*_0_ can develop different Δ*f_t_* values [1,2,3,13,14,15,16]. These results are compared across all four properties in Section 4.5.

### 4.5. Synthesis of the Four PE-ECC Responses

**Figure 18:**
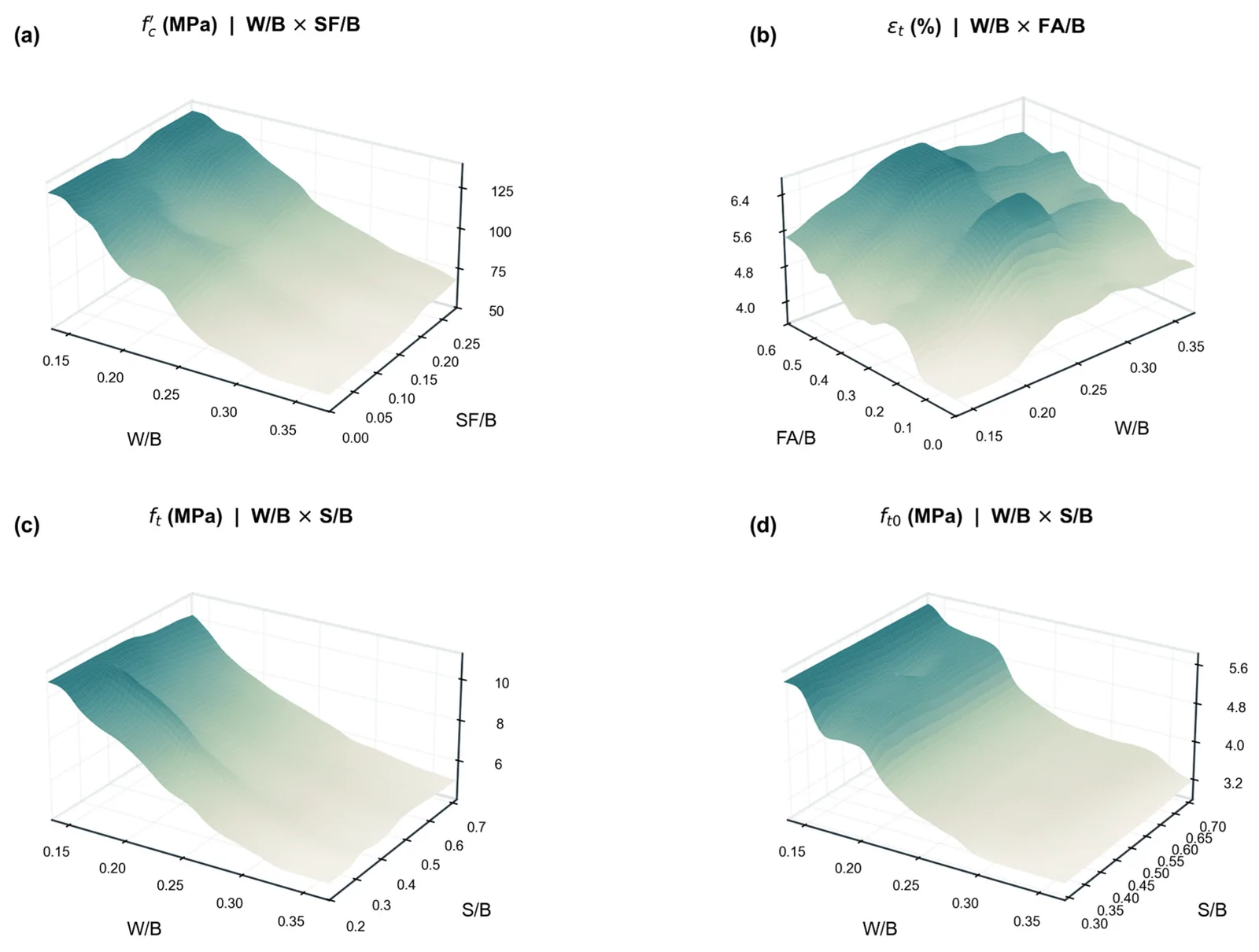
Response surfaces of the four PE-ECC properties: (**a**) $f_{c}^{\prime }$ for W/B × SF/B; (**b**) *ε_t_* for W/B × FA/B; (**c**) *f_t_* for W/B × S/B; (**d**) *f_t_*_0_ for W/B × S/B. Colors represent the fitted response values shown by the color scale in each panel.

Figure 18 brings together four response surfaces for cross-target comparison: W/B-SF/B for $f_{c}^{\prime }$, W/B-FA/B for *ε_t_*, and W/B-S/B for *f_t_* and *f_t_*_0_. The surfaces locate the higher fitted values within the reported PE-ECC mixture range.

The $f_{c}^{\prime }$ surface is dominated by W/B. Its higher-value ridge remains at low W/B in the W/B-SF/B plane, while SF/B mainly changes the height and shape of this low-W/B region. The *ε_t_* surface occupies a different region: higher predicted tensile strain appears closer to moderate W/B and FA-containing binder states, rather than in the lowest-W/B high-strength region. This contrast summarizes the central difference between strength development and tensile deformation in PE-ECC.

Although both *f_t_* and *f_t_*_0_ are higher at low W/B, the *f_t_* surface is more confined to low-to-moderate S/B, whereas *f_t_*_0_ remains broadly aligned with the low-W/B region. The mixture direction associated with peak tensile strength is therefore not identical to that associated with first cracking.

No single mixture direction gives the highest fitted values for all four properties in Figure 18. This trade-off motivates the simultaneous mechanical constraints used for candidate screening in Section 5.

## 5. High-Performance Mixture Features and Constrained Mixture Screening

### 5.1. Reported-Record Benchmark for High-Performance PE-ECC Mixtures

Complete four-target reported records were first used as a benchmark for identifying the high-strain and high-strength PE-ECC region. The benchmark was based on records that simultaneously reported $f_{c}^{\prime }$, *ε_t_*, *f_t_* and *f_t_*_0_, so that high tensile strain and high peak tensile strength could be examined without losing the first-cracking value.

**Figure 19:**
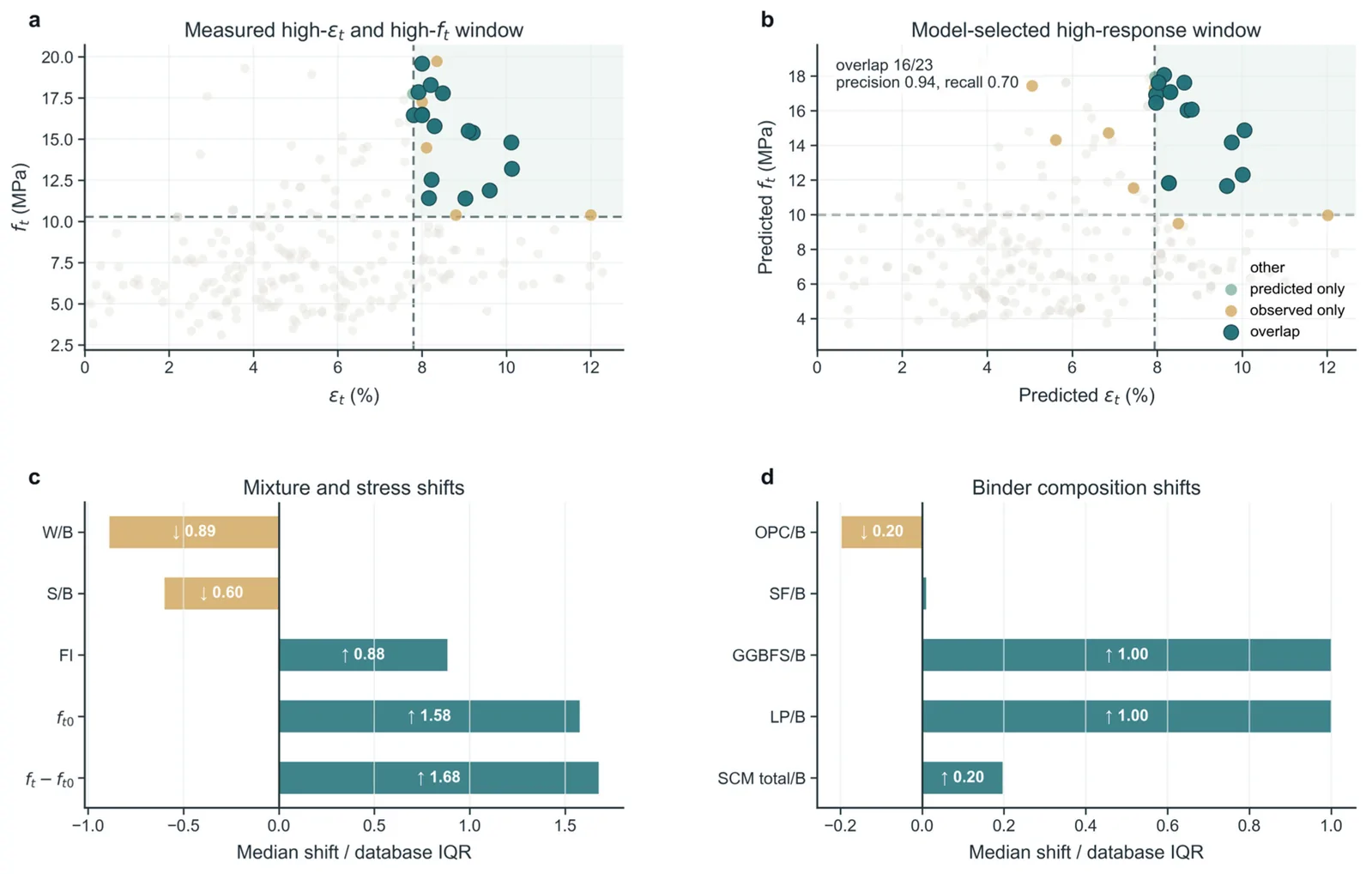
Comparison of regions with high *ε_t_* and *f_t_* in the reported data and model predictions: (**a**) reported records meeting both thresholds; (**b**) predicted records meeting both thresholds and their overlap with the reported set; (**c**) median differences in mixture and tensile variables normalized by the database IQR; (**d**) corresponding differences in binder fractions. Arrows in (**c**,**d**) indicate the direction of the median shift relative to the reported high-response records.

In Figure 19, the high-performance window is defined by the upper-quartile region of reported *ε_t_* and *f_t_*. Twenty-three complete reported records fall in this window. Applying the same *ε_t_*-*f_t_* thresholds to the MoE predictions selected 17 records. Sixteen also lie in the reported window, corresponding to 16/17 of the model-selected set and 16/23 of the reported window. This overlap provides the reported-record benchmark for the subsequent virtual screening.

The high-performance records occupy the upper-right part of the *ε_t_*-*f_t_* field and tend to have lower W/B, lower S/B and higher FI. Their binder compositions generally contain less OPC/B and more GGBFS/B, LP/B and SCM_total_. Here, SCM_total_ is the sum of FA/B, SF/B, GGBFS/B and LP/B. This distribution is compatible with the response maps in Section 4, where higher *ε_t_* occurs more often at lower S/B and greater total binder replacement than in the OPC-rich, low-W/B strength region.

### 5.2. Generation and Filtering of Virtual Candidates Within the Database-Supported Design Space

Virtual candidates were generated around reported PE-ECC records to sample additional mixtures within the supported variable range. A pool of 5000 candidates was produced by perturbing reported mixture records within the prescribed bounds while retaining the reported variable ranges and fiber-material information.

Each virtual point was tied to one parent record with complete reported mixture variables; the parent pool contained 382 records. Binder fractions were perturbed around the parent composition, clipped to non-negative values and renormalized to closure. W/B, S/B, *V_f_*, *L_f_* and *D_f_* were perturbed around the parent values and then clipped to the database Q5-Q95 range. Fiber modulus and fiber tensile strength were inherited from the parent record because these are material properties rather than mixture proportions.

The generation step used three constraints. First, the perturbed binder fractions were renormalized to satisfy the binder-closure condition, OPC/B + FA/B + SF/B + GGBFS/B + LP/B = 1. Second, W/B, S/B, *V_f_*, *L_f_* and *D_f_* were restricted to the database-supported Q5-Q95 range. Third, the generated candidates were filtered by applicability-domain measures in the model feature space. A candidate was retained only when its kNN distance and Mahalanobis distance were below the 95th-percentile support limits calculated from the reported records.

Applicability-domain filtering retained 4000 of the 5000 generated candidates. The support limits were kNN distance ≤ 3.667 and Mahalanobis distance ≤ 5.675, both calculated from the distribution of reported records in the standardized feature space. The retained candidates were evaluated using their predicted mechanical properties, EC, Cost, FI, SCM_total_, Δ*f_t_*, *ρ_t_* and applicability-domain diagnostics.

Nineteen database records and 130 support-filtered virtual mixtures satisfied the mechanical constraints. The virtual set samples additional feasible combinations near reported records without extending the search beyond the stated support limits.

### 5.3. Screening Results and Carbon-Cost Decision Map

The virtual candidates were screened under the same mechanical requirements used for the database-only check. The screening thresholds were set from the reported-record benchmark: *ε_t_* and *f_t_* were required to exceed their reported upper-quartile values, 7.793% and 10.295 MPa, respectively; $f_{c}^{\prime }$ was required to exceed the reported median value, 81.555 MPa. These thresholds were applied to $f_{c}^{\prime }$, *ε_t_* and *f_t_*; Δ*f_t_* > 0 was applied as the fourth screening constraint. *f_t_*_0_ and Δ*f_t_* were also retained as indicators for comparing the feasible candidates:

(7) $$f_{c}^{\prime }\geq f_{c,req}^{\prime },\;\varepsilon_{t}\geq \varepsilon_{t,req},\;f_{t}\geq f_{t,req},\;\Delta f_{t}=f_{t}-f_{t0}>0$$

Here, req denotes the required value for a given screening case. For material-level carbon and cost comparison, the inventory sums were written as

(8) $$EC=\sum_{i}m_{i}e_{i}$$

(9) $$Cost=\sum_{i}m_{i}c_{i}$$

Material-level embodied carbon and material cost are derived indicators rather than measured target properties in the database. In this section, EC denotes material-level embodied carbon calculated from the literature factors, and Cost denotes the corresponding material cost.

The factors listed in Table 4 were adopted from [30,31]. For a one-cubic-meter mixture estimate, ingredient masses can be obtained from reported ratios using a volume-balance calculation and literature densities. The material-level embodied carbon and material cost are then computed using Equations (8) and (9), where *m_i_* is the mass of ingredient *i*, *e_i_* is the unit embodied-carbon factor and *c_i_* is the unit material-cost factor. The represented ingredients are OPC, FA, SF, GGBFS, LP, sand, water and PE fiber. Chemical admixtures are not included because they are not consistently reported.

The comparison is a material-level relative screening. The EC and Cost values are literature-factor-based estimates and do not represent a complete life-cycle assessment or project-specific construction cost. Transportation, mixing, curing, construction, service life and regional cost differences are outside the calculation.

Because EC and Cost are calculated from the literature factors, the carbon-cost ranking should be read with factor sensitivity in mind. Changes in local prices, cement carbon intensity, by-product allocation factors or PE-fiber inventory values can shift the low-carbon and low-cost ranks. For this reason, the mechanical feasibility filter and the carbon-cost ranking are kept separate: the same candidate list can be recalculated with a different factor set without changing the MoE-predicted mechanical properties.

Candidate ranking was performed only within the feasible virtual set. For each candidate, the four mechanical properties were min–max normalized and averaged to obtain the mechanical score. EC and Cost were converted to carbon and cost scores by reversing their normalized values, so that larger scores represented lower EC or Cost. The balanced score was calculated as the average of the mechanical, carbon and cost scores.

Four representative virtual candidates were selected from the feasible set to illustrate different decision preferences: mechanical priority, low carbon, low cost and a balance among mechanical performance, EC and Cost. The candidate for mechanical priority was selected using the mechanical ranking score, the low-carbon and low-cost candidates were selected by the minimum EC and minimum Cost among feasible candidates, and the balanced candidate was selected using a normalized score that combines mechanical performance, EC and Cost. Their values are reported in Table 5.

Table 5 reports the summary variables used to compare the four representative candidates. The table is used for comparing decision preferences rather than for presenting a complete mixture-design specification.

**Figure 20:**
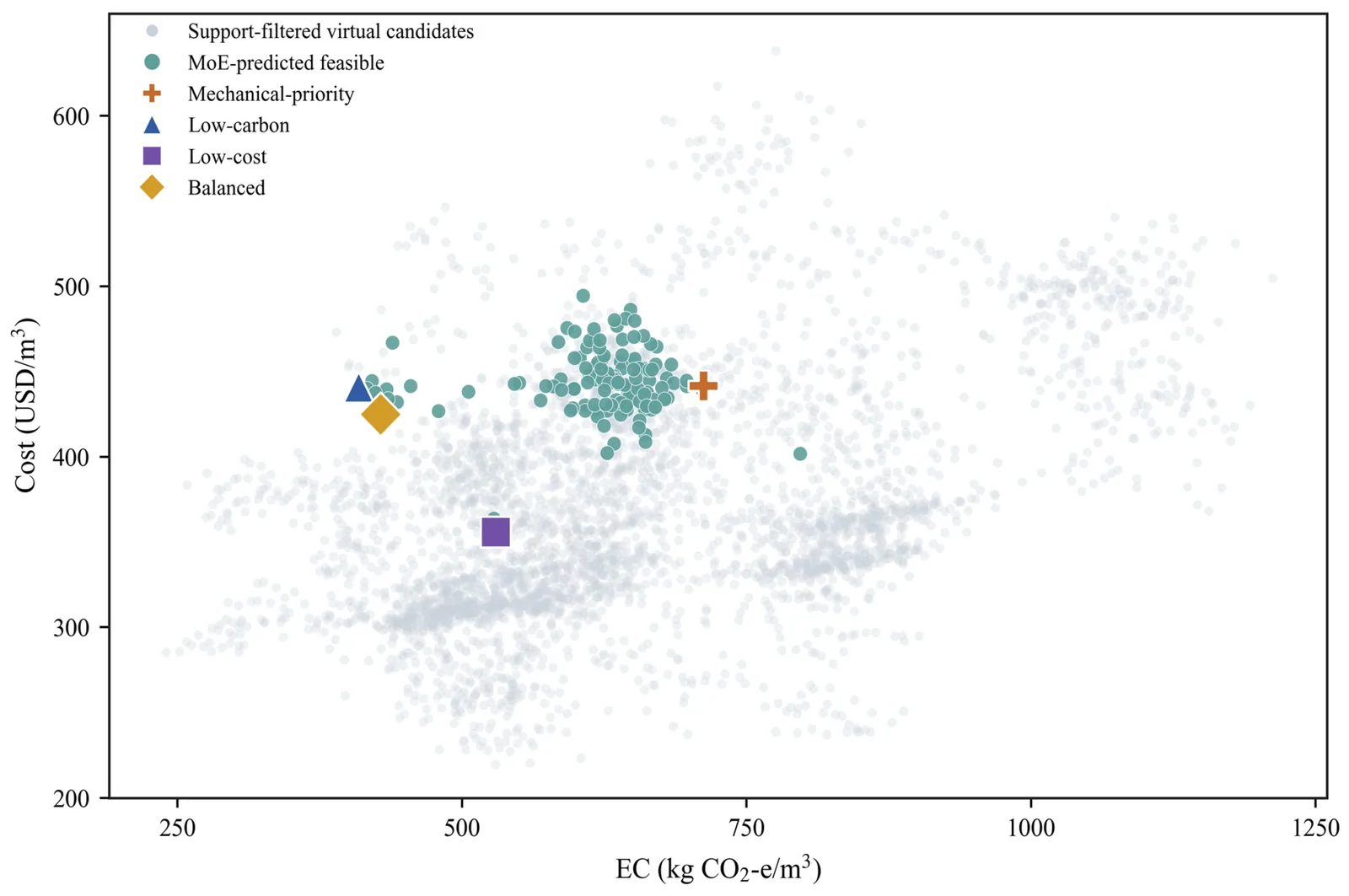
Material-level EC and Cost of the virtual PE-ECC candidates that satisfy the applicability-domain and mechanical criteria.

Figure 20 plots the support-filtered virtual candidates in the EC–Cost plane. Gray points denote all retained virtual candidates, and the highlighted points denote those satisfying the mechanical constraints. The four larger markers identify the representative candidates listed in Table 5. The feasible points occupy a limited part of the retained EC–Cost distribution.

The EC–Cost map shows how the four representative candidates occupy different parts of the decision space. Among the representative candidates, the mechanical-priority option gives the highest predicted $f_{c}^{\prime }$, *ε_t_*, *f_t_* and *f_t_*_0_, but it also has the highest EC. The low-carbon and low-cost candidates occupy different parts of the EC–Cost plane, showing that reducing EC does not necessarily give the lowest material cost. The balanced candidate provides an intermediate choice with predicted *ε_t_* = 8.10%, predicted *f_t_* = 16.06 MPa, EC = 428.3 kg CO_2_-e/m^3^ and Cost = 424.9 USD/m^3^.

### 5.4. Candidate Interpretation and Verification Scope

The representative candidates are used to show how different engineering priorities lead to different mixture choices. The mechanical-priority candidate combines low W/B, low S/B and the highest FI among the four representatives, which is consistent with its high predicted strength and tensile performance. This option is useful when the main objective is to maximize mechanical performance after the feasibility constraints are met, but it does not minimize EC or Cost.

The low-carbon and balanced candidates both have high SCM_total_ values. Their larger total binder replacement lowers the factor-based EC estimate while retaining the predicted tensile requirements. The low-carbon candidate gives EC = 409.1 kg CO_2_-e/m^3^, whereas the balanced candidate gives EC = 428.3 kg CO_2_-e/m^3^ with a higher predicted *f_t_*. The low-cost candidate gives Cost = 355.8 USD/m^3^, but its predicted tensile and compressive strengths are lower than those of the mechanical-priority and balanced candidates. The four candidates thus represent different engineering priorities.

The database-only and virtual screenings give different factor-based candidate ranges. The database-only low-carbon candidate has EC = 580.9 kg CO_2_-e/m^3^, whereas the virtual low-carbon candidate has EC = 409.1 kg CO_2_-e/m^3^. The database-only low-cost candidate has Cost = 405.1 USD/m^3^, whereas the virtual low-cost candidate has Cost = 355.8 USD/m^3^. The lower estimates in the virtual set occur at nearby support-filtered compositions that require laboratory confirmation.

In practical mixture development, the framework moves the preliminary design stage from broad empirical trial and error toward a model-assisted cycle of property prediction, candidate selection, targeted testing and refinement. Once the required property ranges are specified, the predictions and response maps can identify supported mixture regions, compare candidate proportions and guide local optimization before batching. Results from subsequent tests can then be added to the database to update the property models and improve later selections. The framework can therefore serve as an evolving tool for PE-ECC mixture design and analysis, although final proportions must still be adjusted for local materials, workability, curing and test conditions.

The 383-record database covers a broad range of PE-ECC binder, sand and fiber combinations, although record density is not uniform across the four properties. The repeated outer-split and training-fraction analyses show that performance generally stabilizes as more records are included; the larger variation for tensile strain at smaller fractions indicates that this property is more sensitive to sample availability. Curing conditions, specimen geometry, test protocols, fiber orientation and micromechanical quantities were not reported consistently enough to enter the models. Within these boundaries, the response maps show stable fitted trends in the better-supported mixture regions, while the screening results help prioritize targeted experiments. Laboratory tests can verify the representative candidates and extend coverage in sparsely represented regions.

The four candidates are therefore proposed for laboratory verification, not as final optimized designs. Direct tensile tests, crack-pattern measurements, pullout tests and fiber-dispersion characterization are needed to verify whether the predicted balance among strength, tensile ductility, EC and Cost can be achieved experimentally.

## 6. Conclusions

This study used a PE-ECC-only literature database to connect reported binder, sand and fiber variables with four mechanical properties: $f_{c}^{\prime }$, *ε_t_*, *f_t_* and *f_t_*_0_. The fitted mixture-level trends from published PE-ECC records were then used for database-supported mixture screening. The main conclusions are as follows.

1. A PE-ECC-only database containing 383 deduplicated material-level records from 90 verified literature sources was established. Twelve reported binder, sand and fiber variables were used as model inputs. The valid record numbers were 308 for $f_{c}^{\prime }$, 368 for *ε_t_*, 368 for *f_t_* and 304 for *f_t_*_0_. Missing target values were not inferred from other properties, so each target-specific model was trained only with records that reported the corresponding target value.
2. The target-specific MoE stacking ensemble reproduced the four reported PE-ECC properties with high accuracy. The R^2^ values were 0.971, 0.950, 0.970 and 0.954 for $f_{c}^{\prime }$, *ε_t_*, *f_t_* and *f_t_*_0_, respectively. The improvement over the single-model references was larger for the tensile-related targets, for which the reference-model results were less consistent.
3. The response interpretation shows that the four targets follow different mixture trends. $f_{c}^{\prime }$ is mainly associated with low W/B. *ε_t_* does not follow the same densification path and is favored by mixture regions with suitable matrix cracking resistance, paste availability and reported fiber dosage and dimensions. The W/B-S/B maps further show that the high-*f_t_* region is more confined to low-to-moderate S/B, whereas *f_t_*_0_ remains more continuously associated with the low-W/B region.
4. Binder variables should be read as a closed composition rather than as independent SCM dosages. Within this composition, W/B remains the main coordinate for matrix-strength development, while FA-, GGBFS-, SF- and LP-containing states modify the fitted property values through changes in dilution, packing, pozzolanic or latent hydraulic contribution and paste availability. These trends are mixture-level interpretations based on reported variables and require targeted tests for direct verification of hydration, pore structure, fracture behavior and crack-pattern development.
5. The fitted mixture–property relationships and support-filtered screening can be used in an iterative PE-ECC mixture-development process. Predicted properties first narrow the database-supported mixture region, screening identifies candidates for targeted testing, and the resulting test data can be added to the database before the next round of selection. Together, these steps establish a cycle of prediction, screening, testing and model updating, while final proportions still require laboratory verification with local materials.

## Figures and Tables

**Table 1:** Target-specific model performance.

Target	Valid n	R^2^	RMSE	MAE
$f_{c}^{\prime }$	308	0.971	5.952 MPa	1.226 MPa
*ε_t_*	368	0.950	0.562%	0.305%
*f_t_*	368	0.970	0.585 MPa	0.307 MPa
*f_t_*_0_	304	0.954	0.449 MPa	0.239 MPa

**Table 2:** Performance comparison of independent reference models (test-set R^2^).

Model	$f_{c}^{\prime }$	*ε_t_*	*f_t_*	*f_t_*_0_
LSBoost	0.957	0.818	0.956	0.932
GPR	0.893	0.866	0.942	0.947
TabNet	0.831	0.768	0.806	0.856
MoE	0.971	0.950	0.970	0.954

**Table 3:** Reported and predicted properties of mixture M-C.

Property	Reported	Predicted	Absolute Error	Relative Error (%)
$f_{c}^{\prime }$ (MPa)	73.80	79.49	5.69	7.71
*ε_t_* (%)	4.45	4.451	0.001	0.03
*f_t_* (MPa)	9.22	8.84	0.38	4.12

**Table 4:** The literature factors used for material-level EC and Cost estimation in virtual screening.

Component	Symbol	Density (kg/m^3^)	EC Factor (kg CO_2_-e/kg)	Cost Factor (USD/kg)
Ordinary Portland cement	OPC	3130	0.832	0.11
Fly ash	FA	2350	0.009	0.046
Limestone powder	LP	2700	0.017	0.122
Ground granulated blast-furnace slag	GGBFS	2880	0.019	0.10
Silica fume	SF	2170	0.0003	0.50
Sand	S	2640	0.0025	0.014
Water	W	1000	0.0003	0
PE fiber	PE fiber	970	4.08	11.0

**Table 5:** Representative virtual PE-ECC candidates within the database-supported design space.

Preference	W/B	S/B	SCM_total_	FI	$f_{c}^{\prime }$ (MPa)	*ε_t_* (%)	*f_t_* (MPa)	*f_t_*_0_ (MPa)	EC (kg CO_2_-e/m^3^)	Cost (USD/m^3^)
Mechanical priority	0.140	0.31	0.55	18.00	140.2	8.26	19.13	9.81	712.3	441.5
Low carbon	0.148	0.31	0.76	14.98	127.5	7.90	14.39	7.72	409.1	440.8
Low cost	0.184	0.42	0.61	15.00	96.2	7.82	11.43	5.05	529.7	355.8
Balanced	0.140	0.30	0.76	14.91	128.6	8.10	16.06	7.86	428.3	424.9

## Data Availability

The original contributions presented in this study are included in the article/Supplementary Materials. Further inquiries can be directed to the corresponding author.

## Supplementary Materials

The following supporting information can be downloaded at: https://www.mdpi.com/article/10.3390/ma19163363/s1, Data S1: PE-ECC literature dataset used in this study (PE_ECC_Dataset.xlsx).
